# Malaria in pregnancy: Meta-analyses of prevalence and associated complications

**DOI:** 10.1017/S0950268824000177

**Published:** 2024-02-13

**Authors:** Jai K. Das, Sohail Lakhani, Abdu R. Rahman, Faareha Siddiqui, Zahra Ali Padhani, Zainab Rashid, Omar Mahmud, Syeda Kanza Naqvi, Hamna Amir Naseem, Hamzah Jehanzeb, Suresh Kumar, Mohammad Asim Beg

**Affiliations:** 1 Institute for Global Health and Development, Aga Khan University, Karachi, Pakistan; 2 Department of Community Health Sciences, Aga Khan University, Karachi, Pakistan; 3 Robinson Research Institute, Adelaide Medical School, University of Adelaide, Adelaide, Australia; 4 Medical College, Aga Khan University, Karachi, Pakistan; 5 Department of Pathology, Jinnah Sindh Medical University, Karachi, Pakistan; 6 Department of Pathology and Laboratory Medicine, Aga Khan University, Karachi, Pakistan

**Keywords:** epidemiology, malaria, meta-analysis, prevalence

## Abstract

This review aims to assess the prevalence of malaria in pregnancy during antenatal visits and delivery, species-specific burden together with regional variation in the burden of disease. It also aims to estimate the proportions of adverse pregnancy outcomes in malaria-positive women. Based on the PRISMA guidelines, a thorough and systematic search was conducted in July 2023 across two electronic databases (including PubMed and CENTRAL). Forest plots were constructed for each outcome of interest highlighting the effect measure, confidence interval, sample size, and its associated weightage. All the statistical meta-analysis were conducted using R-Studio version 2022.07. Sensitivity analyses, publication bias assessment, and meta-regression analyses were also performed to ensure robustness of the review. According to the pooled estimates of 253 studies, the overall prevalence of malaria was 18.95% (95% CI: 16.95–21.11), during antenatal visits was 20.09% (95% CI: 17.43–23.06), and at delivery was 17.32% (95% CI: 14.47–20.61). The highest proportion of malarial infection was observed in Africa approximating 21.50% (95% CI: 18.52–24.81) during ANC and 20.41% (95% CI: 17.04–24.24) at the time of delivery. Our analysis also revealed that the odds of having anaemia were 2.40 times (95% CI: 1.87–3.06), having low birthweight were 1.99 times (95% CI: 1.60–2.48), having preterm birth were 1.65 times (95% CI: 1.29–2.10), and having stillbirths were 1.40 times (95% CI: 1.15–1.71) in pregnant women with malaria.

## Introduction

### Background

Malaria during pregnancy is a significant source of concern in public health because of the negative repercussions it can have, not only on the mother but also on the developing foetus [1]. According to the World Malaria Report by World Health Organization (WHO), there were 241 million cases of malaria in the year 2020 in 85 malaria endemic countries, an increase from the 227 million cases in 2019 [2]). Concurrently, around 33.8 million pregnancies occurred during the same duration, with 34 percent of women accounting to 11.6 million being exposed to malaria infection during pregnancy [2]).

According to literature, there are two types of malaria that can occur during pregnancy: placental malaria (PM) and gestational malaria (GM), both of which are diagnosed by demonstrating the presence of Plasmodium spp. in the placenta or the mother’s peripheral blood using a thick blood smear (TBS), polymerase chain reaction (PCR), or rapid diagnostic tests [3]. Simple, quick, and more convenient, rapid diagnostic techniques have great potential in malaria detection. They may be of great utility as helpful instruments in the global delivery of health services by improving overall diagnosis of malaria infections. However, the testing procedure must be improved further to overcome the shortcomings of the present implementation. In spite of its drawbacks, such as time and expense, PCR remains the gold standard for identification of malaria parasites [4].

Several unfavourable effects have been reported to occur after parasite sequestration, including maternal anaemia, foetal growth restriction, abortion or stillbirth, premature delivery, and low birthweight (LBW) [5]. Malaria contributes to up to 26% of cases of severe anaemia during pregnancy in endemic regions, and it is responsible for between 0.5 and 23% of all maternal fatalities caused by malaria [6]. In sub-Saharan Africa, malaria during pregnancy is responsible for up to 20% of LBW, or 35% of all avoidable LBW [7, 8]. Successful malaria preventive measures during pregnancy have been shown to reduce perinatal death by 27% [7].

In malaria-endemic regions, pregnancy and the disease have been shown to worsen each other, especially for first-time mothers and individuals who were previously resistant to malaria. Though it has been previously reported that multigravida bear the heaviest burden of malaria in pregnancy both in terms of prevalence and outcome, it is now widely acknowledged that women with greater gravidities, even in areas of low transmission, are also susceptible [7].

About 125 million pregnant women worldwide are at risk of contracting malaria caused by either Plasmodium falciparum or Plasmodium vivax each year [9]. While Plasmodium falciparum malaria is responsible for most of the malaria-related morbidity, Plasmodium vivax may also play a crucial role in certain regions of South America and Southeast Asia [10]. A systematic review of sub-Saharan Africa concluded that the prevalence of Plasmodium falciparum was (22.1%, 95% CI: 17.1–27.2 %), followed by Plasmodium vivax 3% (95%CI: 0–5%), Plasmodium malariae 0.8% (95%CI: 0.3–0.13%), and Plasmodium ovale 0.2% (95%CI: −0.01–0.5) [11]. Similarly, another meta-analysis has shown a significant incidence of malaria in pregnancy in Colombia, which emphasizes the urgent need for researchers, research funding organizations, government agencies, and health authorities to pay more attention to its research and intervention [12].

Based on the significant burden of malaria on the pregnancy outcomes and the health of pregnant women, marked variation in the available evidence is recorded due to diagnostic technique variability, heterogeneity in the enormity of disease, low sample size in some studies, lack of solid meta-analysis of relevant literature, and a substantial lack of understanding on the prevalence of malaria associated in pregnancy, which highlights the significance of a systematic review to quantify the prevalence of disease and understand the underpinnings pertaining to the causality and the burden of outcomes associated. Thus, the current review aims to assess the overall prevalence of malaria in pregnancy along with time-specific burden, that is, during antenatal visits and during delivery and to deduce the specie-specific and regional prevalence of infection. Secondarily, the review also aims to estimate the proportions of adverse pregnancy outcomes and its association with the presence of malarial infection.

## Methods

### Study design

Using the guidelines provided by ‘Preferred Reporting Items for Systematic Reviews and Meta-Analysis (PRISMA)’, a systematic review was conducted. Comprising of a 27-component checklist, the PRISMA guidelines aids in producing a transparent and coherent review which can be easily understood and interpretated globally [13].

### Data source and searches

To find relevant articles, a thorough and systematic search was conducted on 31 July 2023 across two electronic databases (including PubMed and CENTRAL) using precise and accurate search strategies. Publications from the year 2000 to 2023 were searched using database specific strategies. To ensure completeness and entirety, manual searches were also conducted in addition to cross-referencing of source articles to avoid missing out any important source of evidence.

### Search strategies

Based on the MeSH terminologies specific to the objectives and aims of the study, the following search strategy was developed to retrieve studies from databases.

(“Malaria”[Mesh] OR “Malaria, Vivax”[Mesh] OR “Malaria, Falciparum”[Mesh] OR “P. vivax malaria” OR “P. falciparum” OR “maternal malarial” OR “congenital malaria” OR “foetal malaria” OR “malaria in pregnancy” OR “malaria in pregnant”) AND ("Pregnancy"[Mesh:NoExp] OR pregnancy OR pregnant OR “malaria in pregnancy” OR pregnant women OR pregnant woman) AND (parasite densities OR diagnostic test* OR diagnostic* OR endemicity OR Intermittent Preventive Treatment OR IPT OR Intermittent Preventive Therapy OR Insecticide Treated Nets OR drug therapies)

### Eligibility criteria

All the studies quantifying the burden of malaria in pregnancy along with the impact of Plasmodium falciparum and vivax on maternal and child adverse outcomes were taken into consideration. The studies considered eligible were those that were published after the year 2000, were in English language, and catered human subjects only.

The exclusion criteria involved: (1) Clinical trials in which the randomization was done on a predefined criterion; (2) Cohort studies in which the exposure of interest was malaria cases; (3) Case control studies in which the cases were malaria patients as this would not enumerate the burden; (4) Study designs including case reports, case series, commentaries, editorials, narrative reviews, and systematic reviews; (5) Studies using data from previous publications of the author.

To avoid double-counting/the same data being pooled more than once, data reported from different studies, such as those by the same authors, were checked to ensure patient cohorts were non-overlapping.

### Study selection and data extraction

Articles retrieved from the databases were screened by two independent reviewers at a title and abstract level. Articles not immediately ruled out as irrelevant were then reviewed in a similar manner on a full-text basis. Where the relevance of an article was deemed ambiguous, or reviewer decisions conflicted, consensus was reached amongst the authors. Data were then extracted from each included article by a reviewer.

Extracted parameters included author names, publication year, location of study, diagnostic test used for malaria, malaria case count, strain of organism involved, time point in pregnancy at which diagnosis was made, sample size, and calculated prevalence. Additionally, where reported, data were extracted on complications and adverse outcomes for the pregnant women and their foetuses/offspring, for both test-positive and test-negative pregnant women. These data were used to perform secondary analyses to evaluate the association between malaria and maternal and infant morbidity.

Some studies reported adjusted odds ratios but not dichotomized data. Due to the non-uniformity in the method by which these odds ratios were computed, pooling them was deemed invalid and they were not extracted for meta-analysis.

### Studies using multiple diagnostic modalities

Certain included studies tested the same subjects at the time time point for malaria using multiple diagnostic tools. Based on the evidence, a hierarchy of selection was determined to prefer PCR data, followed by microscopy, and then rapid diagnostic tests [13, 14]. In this manner, the most reliable data for a cohort at a given time point were pooled in the analysis without double or triple counting.

### Studies reporting prevalence of multiple strains or at multiple time points

Some included studies did not explicitly state an overall prevalence of malaria but reported prevalence in a strain-wise fashion. In these cases, it was evaluated if the reported patient positive for different strains of malaria were non-overlapping groups. Where this condition was met, the groups were combined, and the overall prevalence was calculated and utilized in the analysis.

Similarly, some studies reported prevalence data for a cohort during ANC and then again during delivery. Given that these estimates were taken at distinct points in time, they were considered separate datapoints and pooled in overall estimates of prevalence.

### Peripheral and placental malaria

Where studies clearly reported overall prevalence data, the data were extracted and analysed simply. However, some studies reported results having tested participants for both peripheral and placental malaria. In such cases, data on peripheral infection were pooled and analysed and placental infection data were only used if that on peripheral infection was not reported.

### Data analysis

The proportions of pregnant women who tested positive for malaria using any diagnostic technique were tabulated. Similarly, the proportions of pregnant women with adverse pregnancy outcomes were also recorded for both test-positive and test-negative women.

Along with confidence intervals of 95%, the following quantitative assessments of malaria were deduced:

1. Overall prevalence of malaria in pregnancy irrespective of the diagnostic test used, period of pregnancy and organism involved.
2. Prevalence of infection during antenatal care and at delivery.
3. Regional disparities of malaria proportions according to UNICEF regions.
4. Association of malaria with adverse pregnancy outcomes.

Due to heterogeneity caused by experimental differences between the included articles, all reported results were computed using a random-effects model meta-analysis. Point estimates and 95% confidence intervals are reported, while heterogeneity was evaluated using the Tau-squared and I-squared metrics, which represent the variance of the distribution of estimates reported by included studies and the percentage of that variation not attributable to sampling error, respectively. Forest plots were constructed for each outcome of interest highlighting the effect measure, confidence interval, sample size, and its associated weightage. Both pooled estimates and sub-groups estimates were illustrated using effective plots.

Publication biases were assessed using DOI plots and LFK index [14]. The sensitivity analysis was conducted through the leave-one-out method. This method recalculates the effect sizes and heterogeneity by removing one study each time [15]. Additionally, meta regression analyses were conducted to evaluate differences in proportions within subgroups of region, species, and diagnostic test.

R-Studio version 2022.07.1 was used to carry out the meta-analysis using the package ‘meta’ (version 6.1.0) [16], and a *p*-value of less than 0.05 was taken as benchmark of significance.

### Quality assessment

Each study included in the systematic review underwent a quality assessment to evaluate the research methodology employed in each study to ensure the reliability and validity of its findings. The Joanna Briggs Institute (JBI) critical appraisal tools, widely acknowledged and reliable for quality assessment, were used to investigate each study [17]. It covers variations of study designs including analytical cross-sectional analysis, case–control, and cohort studies which were used to report the quality of studies in this systematic review. This tool aims to understand the extent to which the study has considered the potential bias in its design and implementation. An overview of the results has been provided in the tables.

## Results

**Figure 1.**
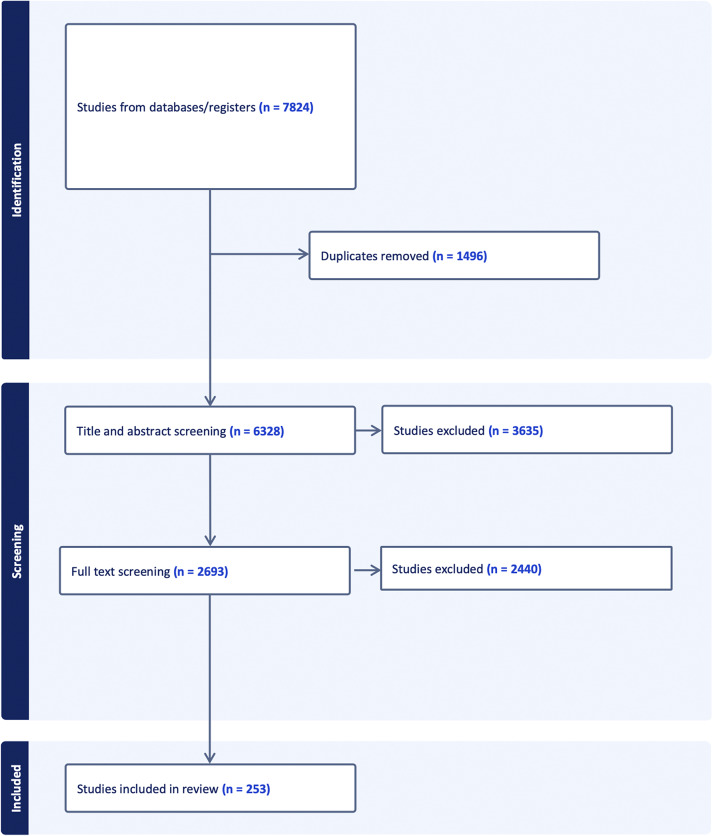
PRISMA diagram of included studies.

Figure 1 below depicts the selection process of the studies included in the review. Initially, 7824 studies were retrieved out of which only 253 qualified for the final inclusion.

The characteristics of the included studies including the author and the year, title, study design, region, sample size, point of pregnancy at which the data were recorded, and diagnostic test used are summarized in Table 1 below.

**Table 1.** Characteristics of included studies

Author/Year	Title	Study design	Region	Sample size	Time point	Diagnostic test
Abdelgadir 2012	Epidemiology of anaemia among pregnant women in Geizera, central Sudan	Cross-sectional	Africa	292	ANC	Microscopy
Abdelrahim 2009	Anaemia, folate and vitamin B12 deficiency among pregnant women in an area of unstable malaria transmission in eastern Sudan	Cross-sectional	Africa	279	ANC	Microscopy
Adam 2005	Prevalence and risk factors for anemia in pregnant women of eastern Sudan	Cross-sectional	Africa	744	ANC	Microscopy
Adam 2007	ABO blood group system and placental malaria in an area of unstable malaria transmission in eastern Sudan	Cross-sectional	Africa	293	Delivery	Microscopy
Adam 2008	Impact of maternal Plasmodium falciparum malaria and hematological parameters on pregnancy and its outcome in southeastern Nigeria	Cross-sectional	Africa	300	ANC	Microscopy
Adam 2009	Placental malaria and lack of prenatal care in an area of unstable malaria transmission in eastern Sudan	Cross-sectional	Africa	236	Delivery	Microscopy
Adam 2012	Decreased susceptibility to placental malaria in anaemic women in an area with unstable malaria transmission in central Sudan	Cross-sectional	Africa	324	Delivery	Microscopy
Adam 2017	Pregnant women carrying female fetuses are at higher risk of placental malaria infection	Cross-sectional	Africa	339	Delivery	Microscopy
Adegnika 2006	Microscopic and sub-microscopic Plasmodium falciparum infection, but not inflammation caused by infection, is associated with low birth weight	Cross-sectional	Africa	145	Delivery	PCR
Adegnika 2010	Epidemiology of parasitic co-infections during pregnancy in Lambare´ne´, Gabon	Cohort	Africa	388	ANC & Delivery	Microscopy
Afutu 2021	High Prevalence of Molecular Markers of Plasmodium falciparum Resistance to Sulphadoxine–Pyrimethamine in Parts of Ghana: A Threat to ITPTp-SP?	Cross-sectional	Africa	3728	ANC & Delivery	PCR
Agu 2013	Impact of Plasmodium falciparum and hookworm infections on the frequency of anaemia in pregnant women of rural communities in Enugu, South-East Nigeria	Cross-sectional	Africa	226	ANC	Microscopy
Agudelo 2013	Prevalence of gestational, placental and congenital malaria in north-west Colombia	Cohort	Latin America and Caribbean	121	ANC & Delivery	PCR
Aguilar 2012	Comparison of placental blood microscopy and the ICT HRP2 rapid diagnostic test to detect placental malaria	Cross-sectional	Africa	1151	Delivery	Microscopy
Aguzie 2017	Antenatal Practices Ineffective at Prevention of Plasmodium falciparum Malaria during Pregnancy in a Sub-Saharan Africa Region, Nigeria	Cross-sectional	Africa	75	ANC	Microscopy
Ahadzie-Soglie 2022	Prevalence and risk factors of malaria and anaemia and the impact of preventive methods among pregnant women: A case study at the Akatsi South District in Ghana.	Cross-sectional	Africa	200	ANC	Microscopy
Ahenkorah 2020	Parasitic infections among pregnant women at first antenatal care visit in northern Ghana: A study of prevalence and associated factors	Cross-sectional	Africa	334	ANC	Microscopy
Ahmed 2014	Placental infections with histologically confirmed Plasmodium falciparum are associated with adverse birth outcomes in India: a cross-sectional study	Cross-sectional	South Asia	506	Delivery	PCR
Ahmed 2015	Performance of four HRP-2/pLDH combination rapid diagnostic tests and field microscopy as screening tests for malaria in pregnancy in Indonesia: a cross-sectional study	Cross-sectional	East Asia and Pacific	950	ANC	PCR
Aliyu 2017	Prevalence, risk factors, and antimalarial resistance patterns of falciparum plasmodiasis among pregnant women in Kaduna metropolis, Nigeria	Cross-sectional	Africa	353	ANC	Microscopy
Almaw 2022	Prevalence of malaria and associated factors among symptomatic pregnant women attending antenatal care at three health centers in north-west Ethiopia.	Cross-sectional	Africa	312	ANC	Microscopy
Akinnawo 2022	Assessing the relationship between gravidity and placental malaria among pregnant women in a high transmission area in Ghana.	Cohort	Africa	1823	Delivery	Placental Biopsy
Anabire 2019a	Prevalence of malaria and hepatitis B among pregnant women in Northern Ghana: Comparing RDTs with PCR	Cross-sectional	Africa	2071	ANC	PCR
Anabire 2019b	Impact of malaria and hepatitis B co-infection on clinical and cytokine profiles among pregnant women	Cross-sectional	Africa	257	ANC	Microscopy
Anabire 2023	High burden of asymptomatic malaria and anaemia despite high adherence to malaria control measures: a cross-sectional study among pregnant women across two seasons in a malaria-endemic setting in Ghana.	Cross-sectional	Africa	269	ANC	PCR
Anchang-Kimbi 2015	Plasmodium falciparum parasitaemia and malaria among pregnant women at first clinic visit in the mount Cameroon Area	Cross-sectional	Africa	303	ANC	Microscopy
Anchang-Kimbi 2017	Profile of red blood cell morphologies and causes of anaemia among pregnant women at first clinic visit in the mount Cameroon area: a prospective cross sectional study	Cross-sectional	Africa	279	ANC	Microscopy
Anchang-Kimbi 2020	Coverage and effectiveness of intermittent preventive treatment in pregnancy with sulfadoxine-pyrimethamine (IPTp-SP) on adverse pregnancy outcomes in the Mount Cameroon area, South West Cameroon	Cross-sectional	Africa	465	Delivery	Microscopy
Appleyard 2008	Malaria in pregnancy in the Solomon islands: barriers to prevention and control	Cross-sectional	East Asia and Pacific	128	ANC	PCR
Ataíde 2010	Using an improved phagocytosis assay to evaluate the effect of HIV on specific antibodies to pregnancy-associated malaria	Cross-sectional	Africa	263	Delivery	Microscopy
Ataíde 2015	Malaria in Pregnancy Interacts with and Alters the Angiogenic Profiles of the Placenta	Cross-sectional	Latin America and Caribbean	137	ANC	Microscopy
Atakorah 2022	Assessment of intestinal and blood protozoan infections among pregnant women visiting ante-natal care at Tafo Hospital, Ghana.	Cross-sectional	Africa	150	ANC	RDT
Avery 2012	Maternal malaria induces a procoagulant and antifibrinolytic state that is embryotoxic but responsive to anticoagulant therapy	Cross-sectional	Africa	193	Delivery	Microscopy
Ayoola 2011	Maternal malaria, birth size and blood pressure in Nigerian newborns: insights into the developmental origins of hypertension from the Ibadan growth cohort	Cohort	Africa	436	ANC & Delivery	Microscopy
Ayoola 2012	Maternal malaria status and metabolic profiles in pregnancy and in cord blood: relationships with birth size in Nigerian infants	Cohort	Africa	467	ANC & Delivery	Microscopy
Ayoya 2006	Determinants of anemia among pregnant women in Mali	Cross-sectional	Africa	131	ANC	Microscopy
Babakhanyan 2016	Influence of Intermittent Preventive Treatment on Antibodies to VAR2CSA in Pregnant Cameroonian Women	Before After	Africa	147	ANC	Microscopy
Bal 2023	Impact of Sub-patent Malaria During Pregnancy on Birth-Weight in Odisha, India: Time-to-Event Analysis of Prospective Longitudinal Follow-Up of a Survey.	Prospective Longitudinal Follow-Up	South Asia	308	ANC	PCR & RDT
Balcha 2023	Prevalence of asymptomatic malaria and associated factors among pregnant women at Boset District in East Shoa Zone, Oromia Region, Ethiopia: a cross-sectional study.	Cross-sectional	Africa	328	ANC	RDT & Microscopy
Bardají 2017	Burden and impact of Plasmodium vivax in pregnancy: A multi-centre prospective observational study	Cohort Study	Latin America and Caribbean	9388	ANC & Delivery	PCR
Bassey 2015	Prevalence of placenta Plasmodium parasitemia and pregnancy outcome in asymptomatic patients at delivery in a university teaching hospital in Nigeria	Cross-sectional	Africa	210	Delivery	Microscopy
Bassiouny 2005	Malaria in late pregnancy in Al Hodeidah Governorate, Yemen	Cross-sectional	Middle East and North Africa	276	Delivery	Microscopy
Bedu-Addo 2014	Reduced prevalence of placental malaria in primiparae with blood group O	Cross-sectional	Africa	827	Delivery	PCR
Benet 2006	Placental malaria in women with South-East Asian ovalocytosis	Cross-sectional	East Asia and Pacific	402	Delivery	Histopathology
Biteghe-Bi-Essone 20222	Intermittent preventive treatment and malaria amongst pregnant women who give birth at the Centre Hospitalier Régional Paul Moukambi de Koula-Moutou in southeastern Gabon.	Transversal Study	Africa	323	Delivery	RDT
Blay 2015	Congenital toxoplasmosis and pregnancy malaria detection post-partum: Effective diagnosis and its implication for efficient management of congenital infection	Cross-sectional	Africa	79	Delivery	PCR
Boel 2010	Complex Interactions between soil-transmitted helminths and malaria in pregnant women on the Thai-Burmese border	Cohort	East Asia and Pacific	490	ANC	Microscopy
Boel 2012	No association of phenotypic ABO blood group and malaria during pregnancy	Cohort	East Asia and Pacific	1468	ANC	Microscopy
Bouyou-Akotet 2003	Prevalence of Plasmodium falciparum infection in pregnant women in Gabon	Cross-sectional	Africa	311	ANC	Microscopy
Bouyou-Akotet 2004	Depressed natural killer cell cytotoxicity against Plasmodium falciparum-infected erythrocytes during first pregnancies	Cross-sectional	Africa	101	Delivery	Microscopy
Bouyou-Akotet 2016	Decrease of microscopic Plasmodium falciparum infection prevalence during pregnancy following IPTp-SP implementation in urban cities of Gabon	Cross-sectional	Africa	387	ANC	Microscopy
Braun 2015	Lack of effect of intermittent preventive treatment for malaria in pregnancy and intense drug resistance in western Uganda	Cross-sectional	Africa	728	Delivery	PCR
Bracho Á 2022	Prevalence of gestational malaria in Ecuador.	Cross-sectional	South America	46	ANC	RDT
Briand 2016	Prevalence of malaria in pregnancy in southern Laos: a cross-sectional survey	Cross-sectional	East Asia and Pacific	536	ANC	PCR
Brutus 2013	Plasmodium vivax malaria during pregnancy, Bolivia	Cross-sectional	Latin America and Caribbean	1003	Delivery	Microscopy
Campos 2011	Diagnosis of gestational, congenital, and placental malaria in Colombia: comparison of the efficacy of microscopy, nested polymerase chain reaction, and histopathology	Cohort	Africa	84	Delivery	PCR
Campos 2012	Plasmodium falciparum infection in pregnant women attending antenatal care in Luanda, Angola	Cross-sectional	Africa	679	ANC	Microscopy
Carmona-Fonseca 2017	Asymptomatic plasmodial infection in Colombian pregnant women	Cohort	Latin America and Caribbean	96	ANC	PCR
Carrara 2013	Malaria burden and artemisinin resistance in the mobile and migrant population on the Thai Myanmar border, 1999-2011: an observational study	Cross-sectional	South Asia	2795	ANC	Microscopy
Cardona-Arias 2022	Frequency of gestational malaria and maternal-neonatal outcomes, in Northwestern Colombia 2009-2020.	Cross- sectional	North America	825	ANC	Microscopy
Cardona-Arias 2023	Diagnostic Accuracy of a Thick Blood Smear Compared to PCR for Malaria Associated with Pregnancy in Colombia.	Cross- sectional	Colombia	829	ANC & Delivery	TBS & PCR
Chaponda 2015	High burden of malaria infection in pregnant women in a rural district of Zambia: a cross-sectional study	Cohort	Africa	1085	ANC	PCR
Cisse 2014	Prevalence and risk factors for Plasmodium falciparum malaria in pregnant women attending antenatal clinic in Bobo-Dioulasso (Burkina Faso)	Cross-sectional	Africa	579	ANC	Microscopy
Cohee 2014	Submicroscopic malaria infection during pregnancy and the impact of intermittent preventive treatment	Cohort	Africa	450	ANC & Delivery	PCR
Corrêa 2017	High burden of malaria and anemia among tribal pregnant women in a chronic conflict corridor in India	Cross-sectional	South Asia	563	ANC	RDT
Cot 2003	Maternally transmitted antibodies to pregnancy-associated variant antigens on the surface of erythrocytes infected with Plasmodium falciparum: relation to child susceptibility to malaria	Cohort	Africa	79	Delivery	Microscopy
Cottrell 2006	Prediction of Plasmodium falciparum placental infection according to the time of infection during pregnanc	Cohort	Africa	281	Delivery	PCR
Cottrell 2015	Submicroscopic Plasmodium falciparum Infections Are Associated With Maternal Anemia, Premature Births, and Low Birth Weight	Cohort	Africa	975	ANC & Delivery	Microscopy
Dechavanne 2015a	Placental Malaria: Decreased Transfer of Maternal Antibodies Directed to Plasmodium falciparum and Impact on the Incidence of Febrile Infections in Infants	Cohort	Africa	535	Delivery	Microscopy
Dechavanne 2015b	Parity-dependent recognition of DBL1X-3X suggests an important role of the VAR2CSA high-affinity CSA-binding region in the development of the humoral response against placental malaria	Cohort	Africa	293	ANC & Delivery	Microscopy
Djontu 2016	Impact of placental Plasmodium falciparum malaria infection on the Cameroonian maternal and neonate’s plasma levels of some cytokines known to regulate T cells differentiation and function	Cross-sectional	Africa	108	Delivery	Microscopy
Doritchamou 2013	Differential adhesion-inhibitory patterns of antibodies raised against two major variants of the NTS-DBL2X region of VAR2CSA	Cross-sectional	Africa	1538	ANC	Microscopy
Dosoo 2020	Epidemiology of malaria among pregnant women during their first antenatal clinic visit in the middle belt of Ghana: a cross sectional study	Cross-sectional	Africa	1655	ANC	Microscopy
Douamba 2012	Asymptomatic malaria correlates with anaemia in pregnant women at Ouagadougou, Burkina Faso	Cross-sectional	Africa	201	ANC	Microscopy
Ebong 2022	Diagnosis of malaria in pregnANC & Othersy: accuracy of CareStart™ malaria Pf/PAN against light microscopy among symptomatic pregnant women at the Central Hospital in Yaoundé, Cameroon.	Cross-sectional	Africa	104	ANC	Microscopy & RDT
Efunshile 2011	Use and effects of malaria control measures in pregnancy in Lagos, Nigeria	Cross-sectional	Africa	400	ANC	PCR
Elbashir 2011	Polymerase chain reaction and histology in diagnosis of placental malaria in an area of unstable malaria transmission in Central Sudan	Cross-sectional	Africa	107	Delivery	PCR
Elghazali 2003	Plasmodium falciparum infection during pregnancy in an unstable transmission area in eastern Sudan	Cohort	Africa	86	ANC	Microscopy
Enato 2009	Plasmodium falciparum malaria in pregnancy: prevalence of peripheral parasitaemia, anaemia and malaria care-seeking behaviour among pregnant women attending two antenatal clinics in Edo State, Nigeria	Cross-sectional	Africa	630	ANC	Microscopy
Esu 2018	Prevalence of the Pfdhfr and Pfdhps mutations among asymptomatic pregnant women in Southeast Nigeria	Cross-sectional	Africa	459	ANC	Microscopy
Fagbemi 2020	Analysis of sulphadoxine-pyrimethamine resistance-associated mutations in Plasmodium falciparum isolates obtained from asymptomatic pregnant women in Ogun State, Southwest Nigeria	Cross-sectional	Africa	406	ANC	PCR
Fairley 2013	Birthweight in offspring of mothers with high prevalence of helminth and malaria infection in coastal Kenya	Cross-sectional	Africa	696	ANC	Microscopy
Fehintola 2012	Intermittent preventive treatment during pregnancy with sulphadoxine-pyrimethamine may promote	Cohort	Africa	306	ANC	Microscopy Plasmodium falciparum gametocytogenesis
Feleke 2020	Asymptomatic malaria infection among pregnant women attending antenatal care in malaria endemic areas of North-Shoa, Ethiopia: a cross-sectional study	Cross-sectional	Africa	263	ANC	Microscopy
Fowkes 2018	Iron deficiency during pregnancy is associated with a reduced risk of adverse birth outcomes in a malaria-endemic area in a longitudinal cohort study	Cohort	East Asia and Pacific	279	ANC & Delivery	Microscopy
Francine 2016	Characterization of asymptomatic Plasmodium falciparum infection and its risk factors in pregnant women from the Republic of Congo	Cross-sectional	Africa	363	ANC	PCR
Fusai 2000	Characterisation of the chondroitin sulphate of Saimiri brain microvascular endothelial cells involved in Plasmodium falciparum cytoadhesion	Cross-sectional	Africa	363	ANC	PCR
Garrison 2022	The Effects of Malaria in Pregnancy on Neurocognitive Development in Children at 1 and 6 Years of Age in Benin: A Prospective Mother-Child Cohort.	Cohort	Africa	493	ANC	PCR
Godwin 2022	Effectiveness of antenatal intermittent preventive treatment for malaria with sulphadoxine-pyrimethamine on peripartum outcomes.	Cross- sectional	Africa	390	ANC & Delivery	Microscopy
Gontie 2020	Prevalence and associated factors of malaria among pregnant women in Sherkole district, Benishangul Gumuz regional state, West Ethiopia	Cross-sectional	Africa	498	ANC	RDT
Griffin 2012	Plasmodium falciparum parasitaemia in the first half of pregnancy, uterine and umbilical artery blood flow, and foetal growth: a longitudinal Doppler ultrasound study	Cohort	Africa	128	ANC	Microscopy
Gutman 2015	The A581G Mutation in the Gene Encoding Plasmodium falciparum Dihydropteroate Synthetase Reduces the Effectiveness of Sulfadoxine-Pyrimethamine Preventive Therapy in Malawian Pregnant Women	Cross-sectional	Africa	1809	Delivery	PCR
Hamann 2010	The toll-like receptor 1 variant S248N influences placental malaria	Cross-sectional	Africa	302	Delivery	Microscopy
Hamer 2009	Burden of malaria in pregnancy in Jharkhand State, India	Cross-sectional	South Asia	3104	ANC & Delivery	RDT
Helegbe 2018	Seroprevalence of Malaria and Hepatitis B Coinfection among Pregnant Women in Tamale Metropolis of Ghana: A Cross-Sectional Study	Cross-sectional	Africa	3127	ANC	RDT
Hounkonnou 2020	Sub-optimal Intermittent Preventive Treatment in pregnancy (IPTp) is associated with an increased risk of submicroscopic P. falciparum infection in pregnant women: a prospective cohort study in Benin	Cohort	Africa	273	ANC	PCR
Hountohotegbe 2020	Circulating Cytokines Associated with Poor Pregnancy Outcomes in Beninese Exposed to Infection with Plasmodium falciparum	Cohort	Africa	400	ANC & Delivery	Microscopy
Huynh 2011	Influence of the timing of malaria infection during pregnancy on birth weight and on maternal anemia in Benin	Cohort	Africa	982	ANC	Microscopy
Ikegbunam 2019	Analysis of Plasmodium falciparum Pfcrt and Pfmdr1 genes in parasite isolates from asymptomatic individuals in Southeast Nigeria 11 years after withdrawal of chloroquine	Cross-sectional	Africa	250	ANC	PCR
Ikegbunam 2022	Malaria surveillance amongst pregnant women attending antenatal care in private hospitals in Onitsha metropolis, South Eastern Nigeria.	Cross-sectional	Africa	270	ANC	Microscopy
Iwalokun 2015	Carriage of Mutant Dihydrofolate Reductase and Dihydropteroate Synthase Genes among Plasmodium falciparum Isolates Recovered from Pregnant Women with Asymptomatic Infection in Lagos, Nigeria	Cross-sectional	Africa	107	ANC	PCR
Jaén-Sánchez 2023 A	Increased peripartum mortality associated with maternal subclinical malaria in Mozambique.	Cross-sectional	Africa	232	Delivery	PCR
Jaén-Sánchez 2023 B	Effects of HIV infection and/or malaria on maternal and neonatal health in a high-prevalence setting.	Cross-sectional	Africa	819	Delivery	RDT
Jäckle 2013	Malaria in pregnancy in rural Gabon: a cross-sectional survey on the impact of seasonality in high-risk groups	Cross-sectional	Africa	1,661	ANC	Microscopy
Jeza 2022	Schistosomiasis, soil transmitted helminthiasis, and malaria co-infections among women of reproductive age in rural communities of Kwale County, coastal Kenya.	Cross-sectional	Africa	534	ANC	Microscopy
Kabanywanyi 2008	Malaria in pregnant women in an area with sustained high coverage of insecticide-treated bed nets	Cross-sectional	Africa	413	Delivery	Microscopy
Kagu 2007	Anaemia in pregnancy: a cross-sectional study of pregnant women in a Sahelian tertiary hospital in Northeastern Nigeria	Cross-sectional	Africa	1040	ANC	Microscopy
Kalilani 2010	The effect of timing and frequency of Plasmodium falciparum infection during pregnancy on the risk of low birth weight and maternal anemia	Cohort	Africa	1172	ANC & Delivery	Microscopy
Kalinjuma 2020	Factors associated with sub-microscopic placental malaria and its association with adverse pregnancy outcomes among HIV-negative women in Dar es Salaam, Tanzania: a cohort study	Cohort	Africa	1115	Delivery	PCR
Kasumba 2000	Low birthweight associated with maternal anaemia and Plasmodium falciparum infection during pregnancy, in a peri-urban/urban area of low endemicity in Uganda	Cross-sectional	Africa	537	Delivery	Microscopy
Kattenberg 2012	Evaluation of antigen detection tests, microscopy, and polymerase chain reaction for diagnosis of malaria in peripheral blood in asymptomatic pregnant women in Nanoro, Burkina Faso	Cross-sectional	Africa	418	ANC	PCR
Kayiba 2021	Evaluation of the usefulness of intermittent preventive treatment of malaria in pregnancy with sulfadoxine-pyrimethamine in a context with increased resistance of Plasmodium falciparum in Kingasani Hospital, Kinshasa in the Democratic Republic of Congo	Cross-sectional	Africa	844	Delivery	Microscopy
Khan 2014	Asymptomatic Plasmodium falciparum malaria in pregnant women in the Chittagong Hill Districts of Bangladesh	Cohort Study	South Asia	526	ANC	PCR
Khattab 2013	Complement activation in primiparous women from a malaria endemic area is associated with reduced birthweight	Cohort	Africa	150	Delivery	Microscopy
King 2021	No evidence of false-negative Plasmodium falciparum rapid diagnostic results in Monrovia, Liberia	Cross-sectional	Africa	87	ANC	PCR
Koladjo 2022	Malaria in the First Trimester of PregnANC & Othersy and Fetal Growth: Results from a Beninese Preconceptional Cohort.	Cohort Study	Africa	218	ANC	Microscopy
Koukouikila-Koussounda 2015	High prevalence of sulphadoxine-pyrimethamine resistance-associated mutations in Plasmodium falciparum field isolates from pregnant women in Brazzaville, Republic of Congo	Cross-sectional	Africa	363	ANC	PCR
Kurth 2010	Adolescence as risk factor for adverse pregnancy outcome in Central Africa--a cross-sectional study	Cross-sectional	Africa	775	Delivery	Microscopy
Lamptey 2019	Association between alpha-thalassaemia trait, Plasmodium falciparum asexual parasites and gametocyte carriage in a malaria endemic area in Southern Ghana	Cohort	Africa	125	ANC	PCR
Lingani 2022	Prevalence and risk factors of malaria among first antenatal care attendees in rural Burkina Faso.	Cross-sectional	Africa	1067	ANC	Microscopy
Liu 2016a	Rapid Diagnostic Test Performance Assessed Using Latent Class Analysis for the Diagnosis of Plasmodium falciparum Placental Malaria	Cross-sectional	Africa	1141	Delivery	Histopathology
Liu 2016b	Diagnosis of placental malaria in poorly fixed and processed placental tissue	Cohort	Africa	182	Delivery	PCR
Lokossou 2013	Association of IL-4 and IL-10 maternal haplotypes with immune responses to P. falciparum in mothers and newborns	Cohort	Africa	576	Delivery	Microscopy
Maïga-Ascofaré 2015	Molecular epidemiology and seroprevalence in asymptomatic Plasmodium falciparum infections of Malagasy pregnant women in the highlands	Cross-sectional	Africa	1244	ANC	PCR
Mama 2022	Intermittent preventive treatment in pregnancy with sulfadoxine-pyrimethamine and parasite resistance & Others: cross-sectional surveys from antenatal care visit and delivery in rural Ghana.	Cross-sectional	Africa	1431	ANC & Delivery	PCR & Microscopy
Manirakiza 2012	Rational case management of malaria with a rapid diagnostic test, Paracheck Pf®, in antenatal health care in Bangui, Central African Republic	Cohort	Africa	452	ANC	RDT
Mankhambo 2002	Evaluation of the OptiMAL rapid antigen test and species-specific PCR to detect placental Plasmodium falciparum infection at delivery	Cross-sectional	Africa	509	Delivery	PCR
Martínez-Pérez 2018	Prevalence of Plasmodium falciparum infection among pregnant women at first antenatal visit in post-Ebola Monrovia, Liberia	Cross-sectional	Africa	195	ANC	PCR
Matangila 2014	Asymptomatic Plasmodium falciparum infection is associated with anaemia in pregnancy and can be more cost-effectively detected by rapid diagnostic test than by microscopy in Kinshasa, Democratic Republic of the Congo	Cross-sectional	Africa	332	ANC	RDT
Mayengue 2004	Submicroscopic Plasmodium falciparum infections and multiplicity of infection in matched peripheral, placental and umbilical cord blood samples from Gabonese women	Cross-sectional	Africa	184	Delivery	PCR
Matambisso 2022	Gravidity and malaria trends interact to modify Plasmodium Falciparum densities and detectability in pregnancy & Others: a 3-year prospective multi-site observational study.	Cohort	Africa	8745	ANC	PCR
Mayor 2009	Sub-microscopic infections and long-term recrudescence of Plasmodium	Cross-sectional	Africa	284	ANC	PCR falciparum in Mozambican pregnant women
Mayor 2018	IgM and IgG against Plasmodium falciparum lysate as surrogates of malaria exposure and protection during pregnancy	Cohort	Africa	207	Delivery	Microscopy
Mbacham 2023	Sub-microscopic Plasmodium Falciparum parasitaemia, dihydropteroate synthase (dhps) resistant mutations to sulfadoxine-pyrimethamine, transmission intensity and risk of malaria infection in pregnancy in Mount Cameroon Region.	Cross-sectional	Africa	874	ANC	PCR & Microscopy
Mbonye 2013	Prescription patterns and drug use among pregnant women with febrile Illnesses in Uganda: a survey in out-patient clinics	Cross-sectional	Africa	998	ANC	Microscopy
Mbouamboua 2019	Sub-microscopic Plasmodium falciparum infections in matched peripheral, placental and umbilical cord blood samples from asymptomatic Congolese women at delivery	Cross-sectional	Africa	370	Delivery	PCR
McClure 2014	A cohort study of Plasmodium falciparum malaria in pregnancy and associations with uteroplacental blood flow and fetal anthropometrics in Kenya	Cohort	Africa	799	ANC & Delivery	PCR
McGready 2004	The effects of Plasmodium falciparum and P. vivax infections on placental histopathology in an area of low malaria transmission	Cohort	East Asia and Pacific	204	ANC	Microscopy
McGready 2012	Adverse effects of falciparum and vivax malaria and the safety of antimalarial treatment in early pregnancy: a population-based study	Cohort	East Asia and Pacific	17613	ANC	Microscopy
McGregor 2017	Obstetric ultrasound aids prompt referral of gestational trophoblastic disease in marginalized populations on the Thailand-Myanmar border	Cohort	East Asia and Pacific	57004	ANC	Microscopy
McLean 2017	P. falciparum infection and maternofetal antibody transfer in malaria-endemic settings of varying transmission	Cohort	East Asia and Pacific	204	Delivery	Microscopy
McLean 2021	High Antibodies to VAR2CSA in Response to Malaria Infection Are Associated With Improved Birthweight in a Longitudinal Study of Pregnant Women	Cohort	East Asia and Pacific	301	ANC & Delivery	Microscopy
Megnekou 2015	Placental malaria and modulation of immune and hormonal responses in Cameroonian women	Cross-sectional	Africa	135	Delivery	Microscopy
Megnekou 2018	Accuracy of One Step malaria rapid diagnostic test (RDT) in detecting Plasmodium falciparum placental malaria infection in women living in Yaoundé, Cameroon	Cross-sectional	Africa	197	Delivery	Microscopy
Minang 2004	Haptoglobin phenotypes and malaria infection in pregnant women at delivery in western Cameroon	Cross-sectional	Africa	119	Delivery	Microscopy
Mlugu 2020	Prevalence and Correlates of Asymptomatic Malaria and Anemia on First Antenatal Care Visit among Pregnant Women in Southeast, Tanzania	Cross-sectional	Africa	819	ANC	PCR
Mockenhaupt 2001	Plasmodium falciparum dihydrofolate reductase alleles and pyrimethamine use in pregnant Ghanaian women	Cross-sectional	Africa	530	ANC	PCR
Mockenhaupt 2002	Diagnosis of placental malaria	Cross-sectional	Africa	596	Delivery	PCR
Mockenhaupt 2003	Reduced prevalence of Plasmodium falciparum infection and of concomitant anaemia in pregnant women with heterozygous G6PD deficiency	Cross-sectional	Africa	529	ANC	PCR
Mockenhaupt 2006	Common polymorphisms of toll-like receptors 4 and 9 are associated with the clinical manifestation of malaria during pregnancy	Cross-sectional	Africa	304	Delivery	Microscopy
Mockenhaupt 2008	Rapid increase in the prevalence of sulfadoxine-pyrimethamine resistance among Plasmodium falciparum isolated from pregnant women in Ghana	Cross-sectional	Africa	530	ANC & Delivery	PCR
Mohammed 2013	Submicroscopic Plasmodium falciparum malaria and low birth weight in an area of unstable malaria transmission in Central Sudan	Case Control	Africa	174	Delivery	Microscopy
Monjol 2017	Detection of Plasmodium falciparum chloroquine resistance transporter (PfCRT) mutant gene amongst malaria-infected pregnant women in Calabar, Nigeria	Case control	Africa	369	ANC	Microscopy
Moore 2016	Safety of artemisinins in first trimester of prospectively followed pregnancies: an observational study	Cross-sectional	East Asia and Pacific	25485	ANC	Microscopy
Moore 2017	Mediation of the effect of malaria in pregnancy on stillbirth and neonatal death in an area of low transmission: observational data analysis	Cross-sectional	East Asia and Pacific	61836	ANC	Microscopy
Mosha 2014	Effectiveness of intermittent preventive treatment with sulfadoxine-pyrimethamine during pregnancy on placental malaria, maternal anaemia and birthweight in areas with high and low malaria transmission intensity in Tanzania	Cohort	Africa	350	Delivery	PCR
Msuya 2011	Anaemia among pregnant women in northern Tanzania: prevalence, risk factors and effect on perinatal outcomes	Cohort	Africa	2654	ANC	Microscopy
Muhangi 2007	Associations between mild-to-moderate anaemia in pregnancy and helminth, malaria and HIV infection in Entebbe, Uganda	Cross-sectional	Africa	2507	ANC	Microscopy
Mukhtar 2006	Congenital malaria among inborn babies at a tertiary centre in Lagos, Nigeria	Cohort	Africa	100	Delivery	Microscopy
Mwin 2021	Predictors of placental malaria in Upper West Regional Hospital-Ghana	Cross-sectional	Africa	300	Delivery	Microscopy
Nacher 2003	Haematinic treatment of anaemia increases the risk of Plasmodium vivax malaria in pregnancy	Cohort	East Asia and Pacific	2112	ANC	Microscopy
Natama 2018	Additional Screening and Treatment of Malaria During Pregnancy Provides Further Protection Against Malaria and Nonmalarial Fevers During the First Year of Life	Cohort	Africa	734	Delivery	PCR
Ndao 2009	Placental malarial infection as a risk factor for hypertensive disorders during pregnancy in Africa: a case-control study in an urban area of Senegal, West Africa	Case control	Africa	490	Delivery	Microscopy
Ndibazza 2013	Associations between maternal helminth and malaria infections in pregnancy and clinical malaria in the offspring: a birth cohort in entebbe, Uganda	Cohort	Africa	2289	ANC	Microscopy
Nega 2015	Prevalence and predictors of asymptomatic malaria parasitemia among pregnant women in the rural surroundings of Arbaminch Town, South Ethiopia	Cross-sectional	Africa	341	ANC	Microscopy
Nekaka 2020	Malaria preventive practices and delivery outcomes: A cross-sectional study of parturient women in a tertiary hospital in Eastern Uganda	Cross-sectional	Africa	210	ANC	Microscopy
Newman 2003	Burden of malaria during pregnancy in areas of stable and unstable transmission in Ethiopia during a nonepidemic year	Cross-sectional	Africa	962	ANC & Delivery	Microscopy
Niang 2008	Accumulation of CVIET Pfcrt allele of Plasmodium falciparum in placenta of pregnant women living in an urban area of Dakar, Senegal	Cross-sectional	Africa	692	Delivery	Microscopy
Nkhoma 2012	The effect of HIV infection on the risk, frequency, and intensity of Plasmodium falciparum parasitemia in primigravid and multigravid women in Malawi	Cohort	Africa	1496	ANC & Delivery	Microscopy
Nlinwe 2022	Impact of long lasting insecticidal nets on asymptomatic malaria during	Cross-sectional	Africa	621	ANC	RDT pregnANC & Othersy, in a rural and urban setting in Cameroon.
Ntoumi 2013	Malaria burden and case management in the Republic of Congo: limited use and application of rapid diagnostic tests results	Cross-sectional	Africa	750	ANC	Microscopy
Nwaefuna 2015	Effectiveness of Intermittent Preventive Treatment in Pregnancy with Sulphadoxine-Pyrimethamine against Submicroscopic falciparum Malaria in Central Region, Ghana	Cross-sectional	Africa	872	ANC	Microscopy
Nyamu 2020	Prevalence and risk factors associated with asymptomatic Plasmodium falciparum infection and anemia among pregnant women at the first antenatal care visit: A hospital based cross-sectional study in Kwale County, Kenya	Cross-sectional	Africa	308	ANC	
Obiri 2020	Histopathological lesions and exposure to Plasmodium falciparum infections in the placenta increases the risk of preeclampsia among pregnant women	Cross-sectional	Africa	134	ANC	Microscopy
Oduwole 2011	Congenital malaria in Calabar, Nigeria: the molecular perspective	Cohort	Africa	204	Delivery	PCR
Ofori 2009	Pregnancy-associated malaria in a rural community of ghana	Cohort	Africa	294	ANC	Microscopy
Ofori 2018	Etiology of Placental Plasmodium falciparum Malaria in African Women	Case control	Africa	807	ANC	Microscopy
Ogbodo 2009	Malaria parasitaemia among pregnant women in a rural community of eastern Nigeria; need for combined measures	Cross-sectional	Africa	272	ANC	Microscopy
Ojurongbe 2011	Prevalence of Dihydrofolate reductase gene mutations in Plasmodium falciparum isolate from pregnant women in Nigeria	Cross-sectional	Africa	179	ANC	Microscopy
Ojurongbe 2018a	Prevalence and associated factors of Plasmodium falciparum and soil transmitted helminth infections among pregnant women in Osun state, Nigeria	Cross-sectional	Africa	200	ANC	Microscopy
Ojurongbe 2018b	High prevalence of dihydrofolate reductase gene mutations in Plasmodium falciparum parasites among pregnant women in Nigeria after reported use of sulfadoxine-pyrimethamine	Cross-sectional	Africa	200	ANC	PCR
Okafor 2006	Risk factors associated with congenital malaria in Enugu, South Eastern Nigeria	Cross-sectional	Africa	625	Delivery	Microscopy
Okoko 2001	Influence of placental malaria infection and maternal hypergammaglobulinaemia on materno-foetal transfer of measles and tetanus	Cross-sectional	Africa	213	Delivery	Microscopy antibodies in a rural west African population
Omer 2011	Submicroscopic and multiple plasmodium falciparum infections in pregnant Sudanese women	Cross-sectional	Africa	836	ANC	PCR
Omer 2017	Placental malaria and its effect on pregnancy outcomes in Sudanese women from Blue Nile State	Cross-sectional	Africa	1149	Delivery	Microscopy
Oraneli 2013	Effect of placental malaria on birth weight of babies in Nnewi, Anambra state, Nigeria	Cross-sectional	Africa	364	Delivery	RDT
Orish 2012	Adolescent pregnancy and the risk of Plasmodium falciparum malaria and anaemia-a pilot study from Sekondi-Takoradi metropolis, Ghana	Cross-sectional	Africa	866	ANC	RDT
Osarfo 2017	Dihydroartemisinin-piperaquine versus artesunate-amodiaquine for treatment of malaria infection in pregnancy in Ghana: an open-label, randomised, non-inferiority trial	Cross-sectional	Africa	3464	ANC	Microscopy
Ouédraogo 2019	Placental impression smears is a good indicator of placental malaria in sub-Saharan Africa	Cross-sectional	Africa	491	Delivery	Microscopy
Oyeyemi 2016	Reliability of rapid diagnostic tests in diagnosing pregnancy and infant-associated malaria in Nigeria	Cross-sectional	Africa	80	ANC	Microscopy
Patel 2016	Absence of Association Between Sickle Trait Hemoglobin and Placental Malaria Outcomes	Cross-sectional	Africa	850	Delivery	PCR
Perrault 2009	Human immunodeficiency virus co-infection increases placental parasite density and transplacental malaria transmission in Western Kenya	Cross-sectional	Africa	157	Delivery	PCR
Pincelli 2018	The Hidden Burden of Plasmodium vivax Malaria in Pregnancy in the Amazon: An Observational Study in Northwestern Brazil	Cohort	Latin America and Caribbean	1180	ANC & Delivery	Microscopy
Plotkin 2014	Placental malaria is rare among Zanzibari pregnant women who did not receive intermittent preventive treatment in pregnancy	Cross-sectional	Africa	1349	Delivery	PCR
Poespoprodjo 2008	Adverse pregnancy outcomes in an area where multidrug-resistant plasmodium vivax and Plasmodium falciparum infections are endemic	Cross-sectional	East Asia and Pacific	3015	Delivery	Microscopy
Poespoprodjo 2011	Highly effective therapy for maternal malaria associated with a lower risk of vertical transmission	Cohort	South Asia	4876	Delivery	Microscopy
Poespoprodjo 2014	Dihydroartemisinin-piperaquine treatment of multidrug resistant falciparum and vivax malaria in pregnancy	Cohort	East Asia and Pacific	6475	ANC	Microscopy
Pujol 2023	Detecting temporal and spatial malaria patterns from first antenatal care visits.	Cross-sectional	Africa	6471	ANC	RDT
Quakyi 2019	High uptake of Intermittent Preventive Treatment of malaria in pregnancy is associated with improved birth weight among pregnant women in Ghana	Cross-sectional	Africa	1922	ANC & Delivery	PCR
Rijken 2012a	Ultrasound evidence of early fetal growth restriction after maternal malaria infection	Cohort	East Asia and Pacific	3779	ANC	Microscopy
Rijken 2012b	Effect of malaria on placental volume measured using three-dimensional ultrasound: a pilot study	Cross-sectional	East Asia and Pacific	84	Delivery	Microscopy
Rogerson 2000a	Malaria and anemia in antenatal women in Blantyre, Malawi: a twelve-month survey	Cross-sectional	Africa	4743	ANC	Microscopy
Rogerson 2000b	Intermittent sulfadoxine-pyrimethamine in pregnancy: effectiveness against malaria morbidity in Blantyre, Malawi, in 1997-99	Cross-sectional	Africa	1623	Delivery	Microscopy
Rogerson 2003a	Diagnosis of Plasmodium falciparum malaria at delivery: comparison of blood film preparation methods and of blood films with histology	Cross-sectional	Africa	464	Delivery	Microscopy
Rogerson 2003b	Placental tumor necrosis factor alpha but not gamma interferon is associated with placental malaria and low birth weight in Malawian women	Cross-sectional	Africa	254	Delivery	Microscopy
Romagosa 2004	Polarisation microscopy increases the sensitivity of hemozoin and Plasmodium detection in the histological assessment of placental malaria	Cross-sectional	Africa	500	Delivery	Microscopy
Ruh 2018	Molecular identification of sulfadoxine-pyrimethamine resistance in malaria infected women who received intermittent preventive treatment in the Democratic Republic of Congo	Cross-sectional	Africa	250	Delivery	Microscopy
Samuels 2022	Diagnostic Performance of Loop-Mediated Isothermal Amplification and Ultrasensitive Rapid Diagnostic Tests for Malaria Screening Among Pregnant Women in Kenya.	Cross-sectional	Africa	482	ANC	RDT & Microscopy
Salifu 2016	Iron Supplementation Alters Heme and Heme Oxygenase 1 (HO-1) Levels In Pregnant Women in Ghana	Cross-sectional	Africa	337	Delivery	PCR
Salih 2011	Monocytes and macrophages and placental malaria infections in an area of unstable malaria transmission in eastern Sudan	Cross-sectional	Africa	93	Delivery	Microscopy
Schmiegelow 2017	Plasmodium falciparum Infection Early in Pregnancy has Profound Consequences for Fetal Growth	Cohort	Africa	157	ANC	RDT
Shannon 2016	Subclinical Plasmodium falciparum infections act as year-round reservoir for malaria in the hypoendemic Chittagong Hill districts of Bangladesh	Cohort	South Asia	589	ANC	Microscopy
Singh 2001	Malaria during pregnancy and infancy, in an area of intense malaria transmission in central India	Cohort	South Asia	274	ANC	Microscopy
Singh 2012	Intervillous macrophage migration inhibitory factor is associated with adverse birth outcomes in a study population in Central India	Case Control	South Asia	4299	Delivery	Microscopy
Singh 2014	Placental and neonatal outcome in maternal malaria	Cohort	South Asia	203	ANC	Microscopy
Singh 2020	Association of Angiopoietin Dysregulation in Placental Malaria with Adverse Birth Outcomes	Cross-sectional	South Asia	7873	Delivery	Microscopy
Sirima 2003	Failure of a chloroquine chemoprophylaxis program to adequately prevent malaria during pregnancy in Koupéla District, Burkina Faso	Cross-sectional	Africa	597	ANC & Delivery	Microscopy
Sohail 2015	Prevalence of Malaria Infection and Risk Factors Associated with Anaemia among Pregnant Women in Semiurban Community of Hazaribag, Jharkhand, India	Cross-sectional	South Asia	2141	ANC & Delivery	Microscopy
Soulard 2011	Placental malaria-associated suppression of parasite-specific immune response in neonates has no major impact on systemic CD4 T cell homeostasis	Cross-sectional	Africa	54	Delivery	Microscopy
Stanisic 2015	Risk factors for malaria and adverse birth outcomes in a prospective cohort of pregnant women resident in a high malaria transmission area of Papua New Guinea	Cohort	East Asia and Pacific	328	ANC & Delivery	PCR
Stephens 2014	Prevalence of peripheral blood parasitaemia, anaemia and low birthweight among pregnant women in a suburban area in coastal Ghana	Cohort	Africa	320	ANC	Microscopy
Strand 2003	Infectious aetiology of jaundice among pregnant women in Angola	Case Control	Africa	60	ANC	Microscopy
Subussa 2021	Asymptomatic Plasmodium infection and associated factors among pregnant women in the Merti district, Oromia, Ethiopia	Cross-sectional	Africa	364	ANC	Microscopy
Sylvester 2016	Prenatal exposure to Plasmodium falciparum increases frequency and shortens time from birth to first clinical malaria episodes during the first two years of life: prospective birth cohort study	Cohort	Africa	206	Delivery	Microscopy
Tahita 2013	Clinical signs and symptoms cannot reliably predict Plasmodium falciparum malaria infection in pregnant women living in an area of high seasonal transmission	Case Control	Africa	600	ANC	RDT
Tako 2005	Risk factors for placental malaria and its effect on pregnancy outcome in Yaounde, Cameroon	Cross-sectional	Africa	1895	Delivery	Microscopy
Taylor 2017	Minimal Impact by Antenatal Subpatent Plasmodium falciparum Infections on Delivery Outcomes in Malawian Women: A Cohort Study	Cohort	Africa	923	ANC	PCR
Teo 2014	Decreasing malaria prevalence and its potential consequences for immunity in pregnant women	Cohort	Africa	744	ANC	Microscopy
Toure 2014	Coverage and efficacy of intermittent preventive treatment with sulphadoxine pyrimethamine against malaria in pregnancy in Côte d’Ivoire five years after its implementation	Cross-sectional	Africa	1317	Delivery	Microscopy
Tran 2020	The impact of gravidity, symptomatology and timing of infection on placental malaria	Cohort	Africa	275	Delivery	Microscopy
Ugwu 2014	Malaria and anaemia in pregnancy: a cross-sectional study of pregnant women in rural communities of Southeastern Nigeria	Cross-sectional	Africa	300	ANC	Microscopy
Ukaga 2007	Placental malaria in Owerri, Imo State, south-eastern Nigeria	Cross-sectional	Africa	586	Delivery	Microscopy
Uneke 2007	Impact of maternal Plasmodium falciparum malaria and haematological parameters on pregnancy and its outcome in southeastern Nigeria	Cross-sectional	Africa	300	ANC	Microscopy
Unger 2019	Microscopic and submicroscopic Plasmodium falciparum infection, maternal anaemia and adverse pregnancy outcomes in Papua New Guinea: a cohort study	Cohort	East Asia and Pacific	2190	ANC & Delivery	Microscopy
Unger 2022	Associations of maternal iron deficiency with malaria infection in a cohort of pregnant Papua New Guinean women.	Cohort	East Asia and Pacific	1888	ANC & Delivery	Microscopy
Valente 2011	Prevalence and risk factors of Plasmodium falciparum infections in pregnant women of Luanda, Angola	Cross-sectional	Africa	567	Delivery	PCR
vanEijk 2001	Human immunodeficiency virus seropositivity and malaria as risk factors for third-trimester anemia in asymptomatic pregnant women in western Kenya	Cross-sectional	Africa	4608	ANC	Microscopy
vanEijk 2009	Geohelminth Infections among pregnant women in rural western Kenya; a cross-sectional study	Cross-sectional	Africa	673	ANC	Microscopy
VanGeertruyden 2005	Malaria infection among pregnant women attending antenatal clinics in six Rwandan districts	Cross-sectional	Africa	1432	ANC	Microscopy
vanLenthe 2019	Markers of sulfadoxine-pyrimethamine resistance in Eastern Democratic Republic of Congo; implications for malaria chemoprevention	Cross-sectional	Africa	514	ANC	PCR
vanLoon 2019	MiRNA-146a polymorphism increases the odds of malaria in pregnancy	Cross-sectional	Africa	509	ANC & Delivery	PCR
Vásquez 2018	Performance of a highly sensitive rapid diagnostic test (HS-RDT) for detecting malaria in peripheral and placental blood samples from pregnant women in Colombia	Cohort	Latin America and Caribbean	737	ANC & Delivery	Microscopy
Vásquez 2020a	Evaluation of highly sensitive diagnostic tools for the detection of P. falciparum in pregnant women attending antenatal care visits in Colombia	Cross-sectional	Latin America and Caribbean	858	ANC	PCR
Verhoeff 2004	Post-neonatal infant mortality in Malawi: the importance of maternal health	Cohort	Africa	451	ANC & Delivery	Microscopy
Walther 2010	Placental malaria is associated with reduced early life weight development of affected children independent of low birth weight	Cohort	Africa	783	Delivery	Microscopy
Williams 2016	Non-falciparum malaria infections in pregnant women in West Africa	Cross-sectional	Africa	2526	ANC	RDT
Woodburn 2009	Risk factors for helminth, malaria, and HIV infection in pregnancy in Entebbe, Uganda	Cross-sectional	Africa	2507	ANC	Microscopy
Wumba 2015	Interactions between malaria and HIV infections in pregnant women: a first report of the magnitude, clinical and laboratory features, and predictive factors in Kinshasa, the Democratic Republic of Congo	Cross-sectional	Africa	332	ANC	Microscopy
Yatich 2010	Malaria and intestinal helminth co-infection among pregnant women in Ghana: prevalence and risk factors	Cross-sectional	Africa	746	ANC	Microscopy
Yeboah 2016	Quality of Sulfadoxine-Pyrimethamine Given as Antimalarial Prophylaxis in Pregnant Women in Selected Health Facilities in Central Region of Ghana	Cross-sectional	Africa	543	ANC	Microscopy
Yovo 2022	Assessing fetal growth in Africa: Application of the international WHO and INTERGROWTH-21st standards in a Beninese cohort.	Cohort	Africa	411	ANC	RDT
Zablon 2015	Prevalence of Plasmodium falciparum Malaria among Pregnant Students in Dodoma Region, Tanzania: No Cases Have Been Detected	Cross-sectional	Africa	50	ANC	Microscopy
Zhou 2002a	Prevalence of Plasmodium falciparum infection in pregnant Cameroonian women	Cohort	Africa	719	ANC	Microscopy

## Prevalence trends

Supplementary Figure 2 shows overall trends of prevalence of malaria in an ascending order of years, estimated from 253 studies. As evident, the proportions have remained relatively persistent with the passing years and no significant reduction has been observed from the year 2000 to year 2023.

According to the pooled estimates, the prevalence of malaria was 18.95% (95% CI: 16.95–21.11, n=375,792) based on random-effects model. Similarly, when bifurcated on the time of reporting, the prevalence of malaria during antenatal visits was 20.09% (95% CI: 17.43–23.06, n =282,169, studies = 182) and during delivery was 17.32% (95% CI: 14.47–20.61, n = 93,623, studies = 121) using the same random-effects model. The heterogeneity was deduced using I-squared test, which was reported to be 99% in each model. Sensitivity analysis showed no change in the heterogeneity (Supplementary Appendix Figure 1a). The DOI plot was symmetrical indicating no publication bias (Supplementary Appendix Figure 1b).

### Specie-specific prevalence

**Figure 2.**
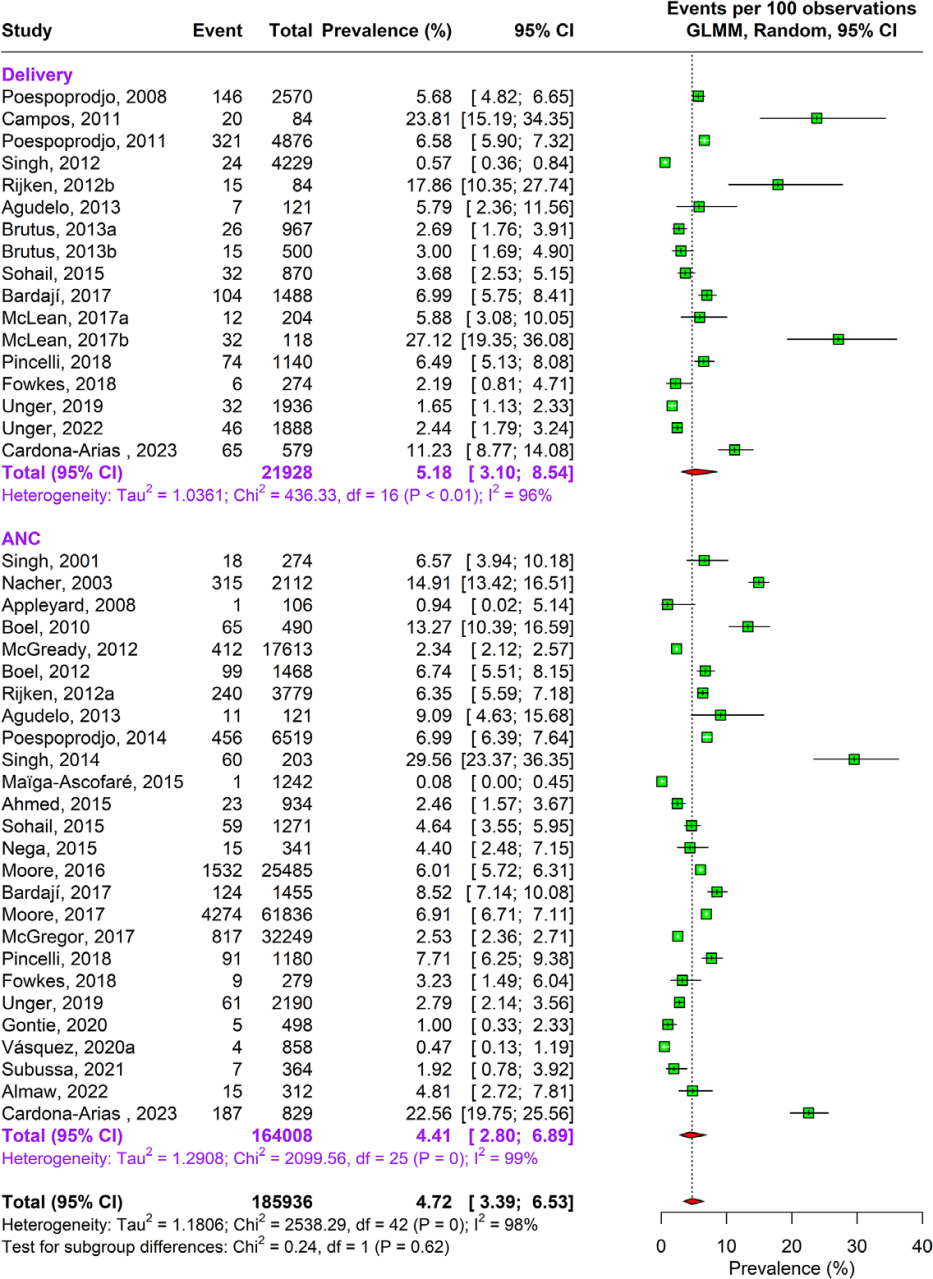
Forest Plot depicting Plasmodium vivax pooled estimates of prevalence of malaria with 95% CIs.

**Figure 3.**
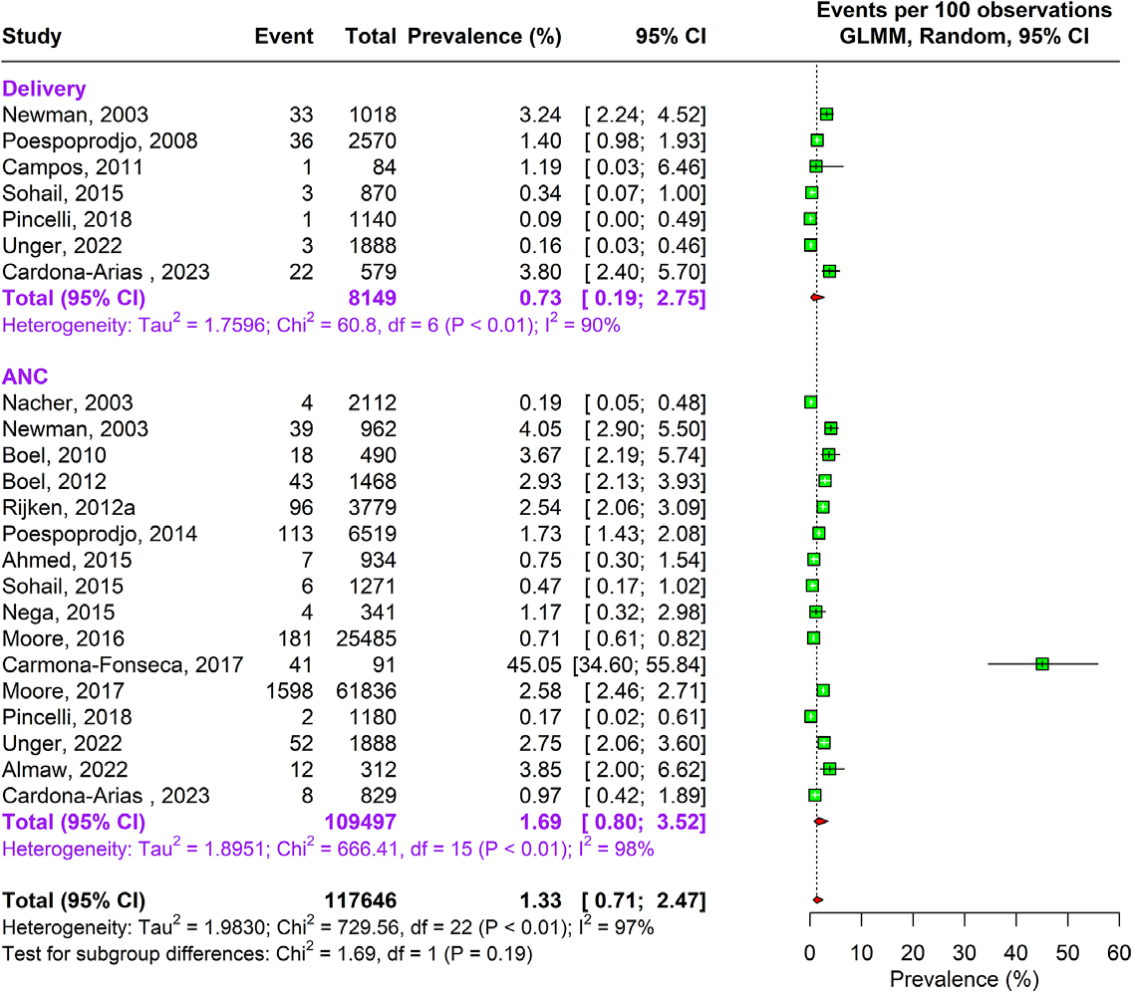
Forest Plot depicting Plasmodium falciparum and vivax pooled estimates of prevalence of malaria with 95% CIs.

During the antenatal period, the prevalence of malaria caused by Plasmodium falciparum alone was 17.76% (95% CI: 15.04–20.85, n = 269,537, studies = 166) using random-effects model. This was followed by Plasmodium vivax caused infections accounting to 4.41% (95% CI: 2.80–6.89, n = 164,008, studies = 26) prevalence. In about 1.69% (95% CI: 0.80–3.52, n = 109,497, studies = 16) pregnant women, traces of both Plasmodium falciparum and vivax species were found as shown in Supplementary Figure 3a and Figures 2 and 3.

A similar pattern of infection was observed during delivery. Approximately 16.55% (95% CI: 13.57–20.04, n= 73,417, studies = 113) pregnant women were infected by Plasmodium falciparum and 5.18% (95% CI: 3.10–8.54, n= 21,928 studies = 17) by Plasmodium vivax, and 0.73% (95% CI: 0.19–2.75, n = 8149, studies = 7) were infected by both Plasmodium falciparum and vivax. The sensitivity analysis showed no change in heterogeneity (Supplementary Appendix Figure 3a–c). The DOI plots showed no asymmetry for Plasmodium falciparum but for Plasmodium vivax alone and combined vivax and falciparum thus concluding positive publication bias (Supplementary Appendix Figure 2a–c).

### Regional distribution of malarial infection

The meta-analysis revealed that the highest proportion of malarial infection during ANC was observed in Africa approximating 21.50% (95% CI: 18.52–24.81, n = 110,012, studies = 143). This was followed East Asia and Pacific region accounting to 17.28% (95% CI: 9.29–29.86, n = 157,986, studies = 18). The lowest prevalence was observed in South Asia 8.66% (95% CI: 3.06–22.17, n = 8,513, studies = 9) followed by Latin America and Caribbean region 14.20% (95% CI: 6.31–28.91, n = 3,929, studies = 7) as shown in Supplementary Figure 4a. Sensitivity analysis revealed no significant difference. A symmetrical DOI plot was also indicative of no publication bias (Supplementary Appendix Figures 4a and 5a).

A similar random-effects meta-analysis at the time of delivery revealed that the prevalence of malaria in Africa was 20.41% (95% CI: 17.04–24.24, n = 46,925, studies = 95), in East Asia in Pacific Region was 16.33% (95% CI: 8.46–29.19, n = 22,214, studies =12), in Latin America and Caribbean region was 5.28% (95% CI: 2.68–10.12, n = 4,834, studies = 7), and in South Asia was 4.14% (95% CI: 1.52–10.80, n = 19,071, studies = 6) as shown in Supplementary Figure 4b. Sensitivity analysis revealed no significant difference. On the other hand, DOI for delivery showed minor asymmetry favouring positive publication bias (Supplementary Appendix Figure 5b).

## Associations with prevalence of malaria

Adverse pregnancy outcomes have shown mild-to-moderate associations with the prevalence of malarial infection in pregnancy.

### Anaemia

**Figure 4.**
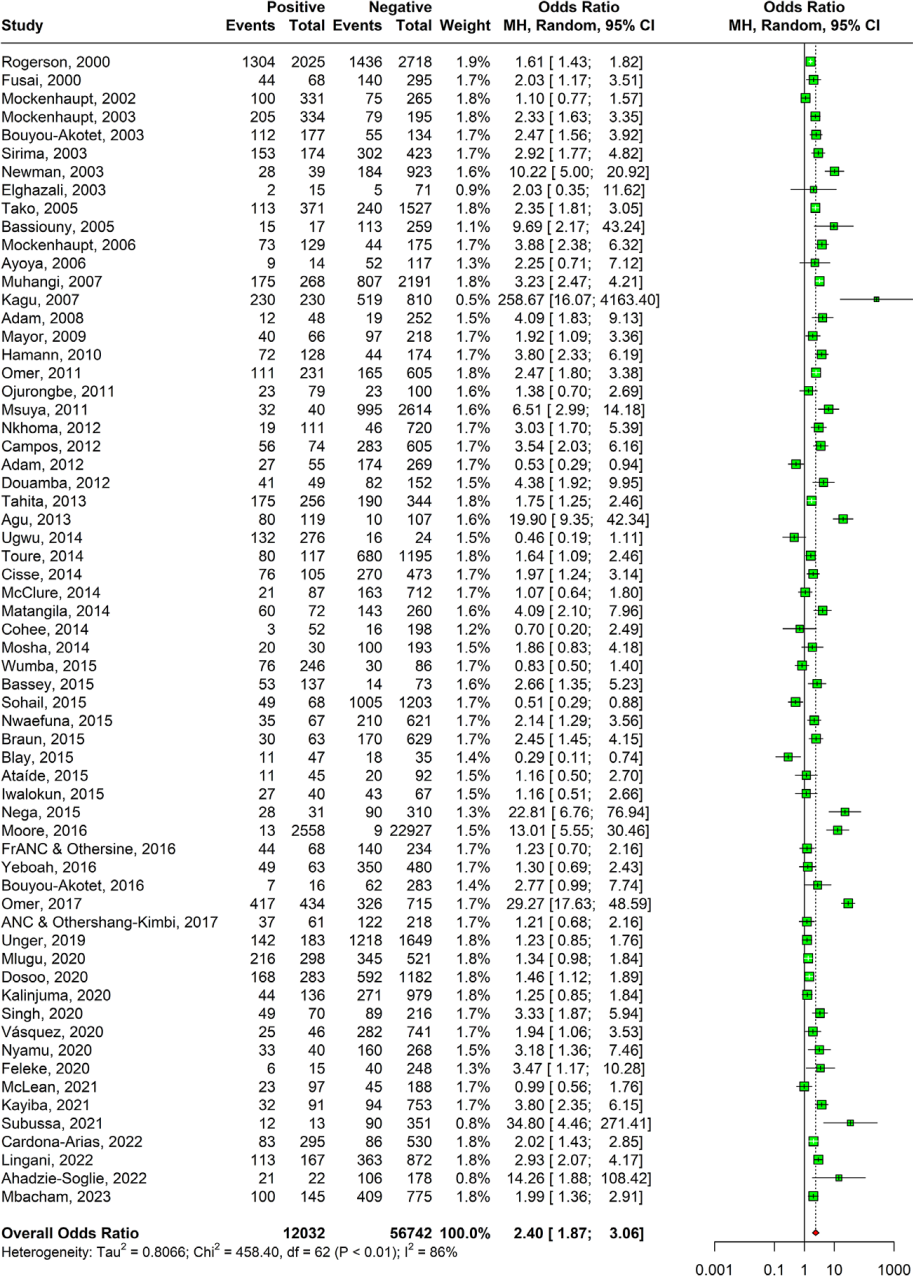
Forest plot confirming association of malaria in pregnancy and anaemia.

A statistically significant association was observed between anaemia and malaria presence in 62 studies as shown in Figure 4. The odds of having anaemia were 2.40 times (95% CI: 1.87–3.06) in malaria-positive women as compared to malaria-negative women. The heterogeneity of the studies as calculated with I-squared value was 86%. Sensitivity analysis revealed that the effect size of meta-analysis was deviating significantly due to two studies; hence, they were excluded (Supplementary Appendix Figure 6). The DOI plot showed minor asymmetry thus depicting minimal publication bias (Supplementary Appendix Figure 7).

### Low birthweight

**Figure 5.**
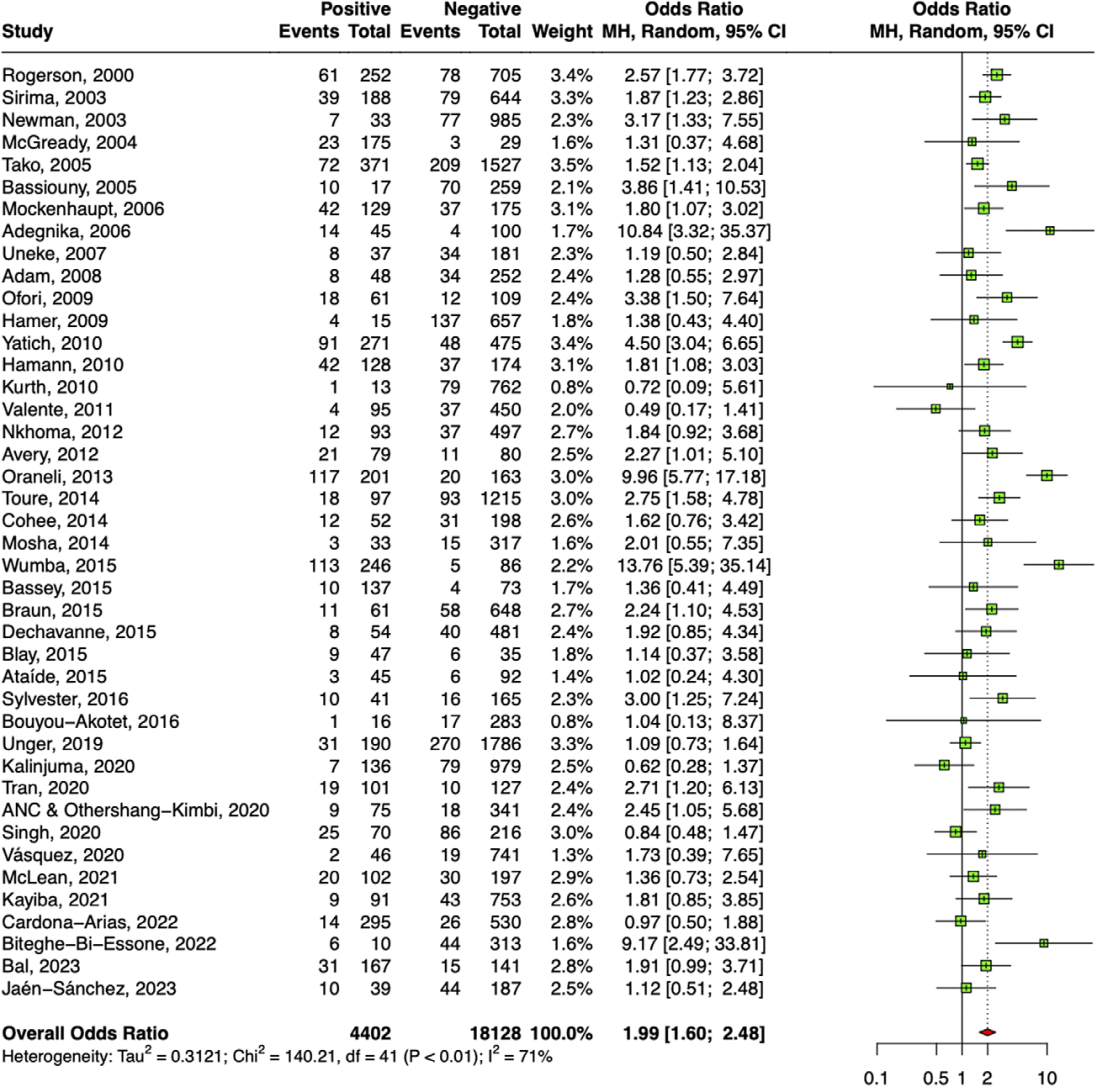
Forest plot confirming association of malaria in pregnancy and LBW.

A significant association of low birthweight of the babies and malaria-positive women was also observed after pooling estimates from 42 studies as shown in Figure 5. The overall odds ratio deduced was 1.99 (95% CI: 1.60–2.48). Sensitivity analyses revealed that two studies were responsible for major deviation in the effect size; hence, they were excluded. Absence of publication bias was confirmed by symmetrical DOI plot (Supplementary Appendix Figure 9).

### Pre-term birth

**Figure 6.**
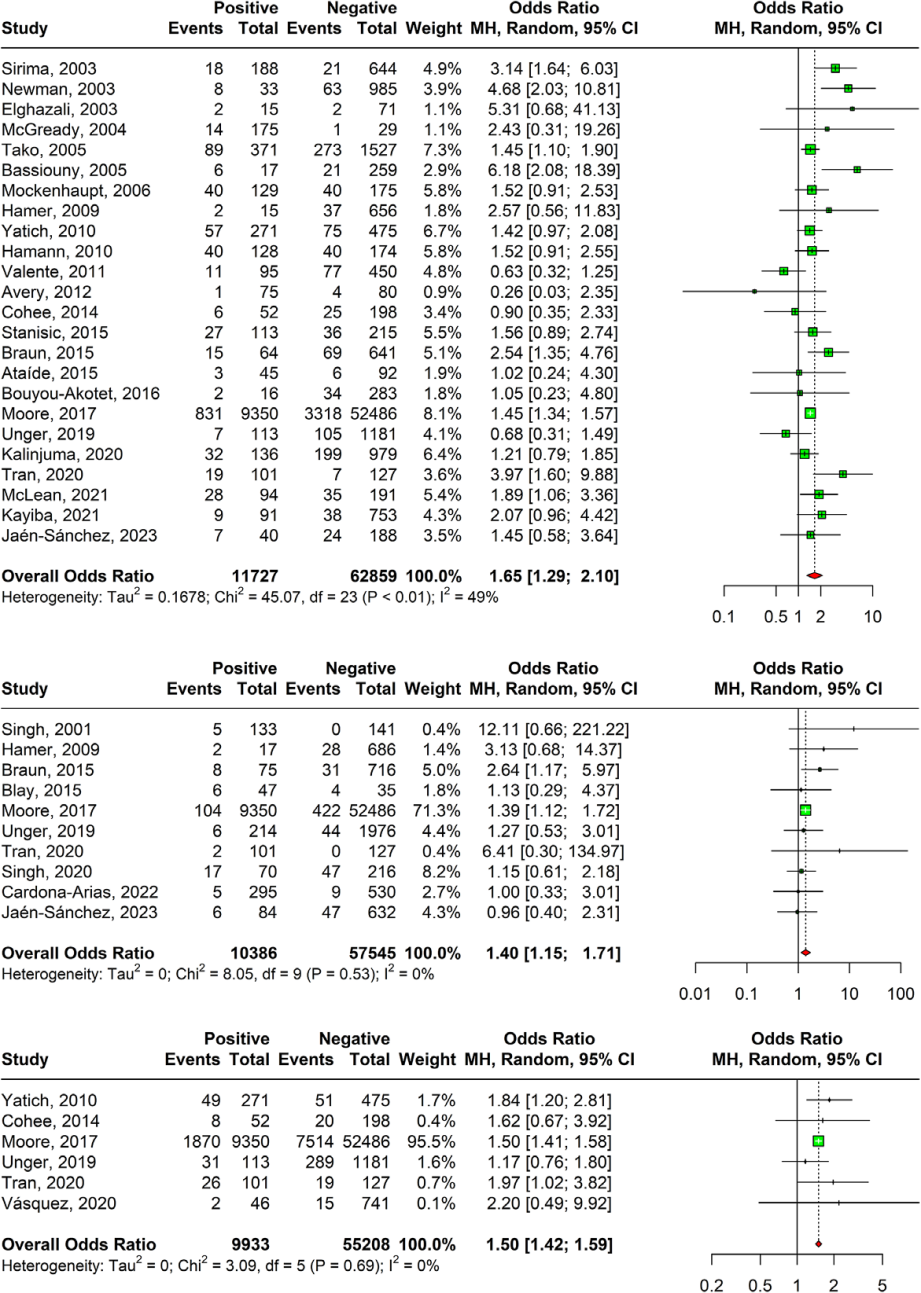
(a) Forest plot confirming association of malaria in pregnancy and preterm births. (b) Forest plot confirming association of malaria in pregnancy and stillbirths. (c) Forest plot confirming association of malaria in pregnancy and SGA.

A positive relation between malaria in pregnancy and preterm births was observed in 24 studies with an overall odds ratio of 1.65 (95% CI: 1.29–2.10) as shown in Figure 6. The random-effects model took into consideration the heterogeneity of 49% as calculated by I-squared value. Sensitivity analysis revealed that the effect size of meta-analysis was deviating significantly due to one study; hence, it was excluded. The DOI plot showed major asymmetry, thus indicating positive publication bias (Supplementary Appendix Figure 11).

### Stillbirth

A statistically significant association was observed between stillbirths amongst malaria test-positive pregnant women with and odds ratio of 1.40 (95% CI: 1.15–1.71) based on ten studies as shown in Figure 6b. Sensitivity analyses revealed that one study was responsible for major deviation in the effect size; hence, it was excluded. The DOI plot showed major asymmetry, thus indicating positive publication bias (Supplementary Appendix Figure 13).

### Small for gestational age (SGA)

A significant association has been observed between SGA and pregnancy malaria with an overall odds ratio of 1.50 (95% CI: 1.42–1.59) 1.39 (95% CI: 0.99–1.96) using estimates of six studies as shown in Figure 6c. Sensitivity analysis revealed that the effect size of meta-analysis was deviating significantly due to one study; hence, it was excluded. The DOI plot shows minor asymmetry, thus depicting minimal publication bias (Supplementary Appendix Figure 15).

### Abortion

An insignificant statistical association was observed in abortion and malaria in pregnancy with an odds ratio of 0.85 (95% CI: 0.21–3.48) using estimates from five studies (Supplementary Appendix Figure 16). Sensitivity analyses revealed that two studies were responsible for major deviation in the effect size; hence, they were excluded. The DOI plot showed major asymmetry, thus confirming negative publication bias (Supplementary Appendix Figure 17).

### Preeclampsia

A statistically insignificant association was seen with pre-eclampsia using the estimates from three studies with an odds ratio of 0.82 (95% CI: 0.16–4.34). Sensitivity analyses revealed that one study was responsible for major deviation in the effect size; hence, it was excluded (Supplementary Appendix Figure 18). The DOI showed no asymmetry, thus confirming absence of publication bias (Supplementary Appendix Figure 19).

### Growth restriction

A statistically insignificant association was seen with growth restriction using the estimates from two studies with an odds ratio of 1.21 (95% CI: 0.04–35.52, n= 508). There was no change in effect observed during sensitivity analysis (Supplementary Appendix Figure 20). The DOI showed major asymmetry, thus confirming negative publication bias (Supplementary Appendix Figure 21).

## Meta regression

Results of meta regression analyses for region, diagnostic test, and specie variables are displayed in Table 2 Test of moderators were found significant in both region (*p* < 0.001) and specie (*p*-value < 0.01), indicating a significant influence on the effect sizes. The R-squared for region showed that 10.45% of the difference in the true effect sizes can be explained by the region, and 3.67% by the specie, and 1.22% by the diagnostic variable.

**Table 2.** Meta regression analysis of effect size with respect to region, diagnostic tests, and specie

Sub-group	Estimate	SE	*p*-value	CI (95%)
Region				
South Asia (Reference)				
Africa	1.3527	0.3033	<.0001	1.9496, 0.7558
East Asia and the Pacific	1.0741	0.3601	0.0031	1.7826, 0.3655
Latin America and the Caribbean	0.3279	0.425	0.441	1.1643, −0.5085
South America	1.9233	0.562	0.0007	3.0292, 0.8173
Diagnostic Test				
Histopathology (Reference)				
PCR	−0.3059	0.6953	0.6603	−1.6742, 1.0624
Microscopy	−0.5356	0.6889	0.4375	−1.8913, 0.8201
RDT	−0.6884	0.7323	0.348	−2.1296, 0.7528
Specie				
Plasmodium Vivax (Reference)				
Plasmodium falciparum	0.4289	0.16	0.01	0.0945, 0.7632
Plasmodium falciparum and Vivax	−0.8248	0.6073	0.175	−2.0199, 0.3703

For meta-regression analysis by region, South America had the highest effect sizes when compared with South Asia (b=1.92, *p* < 0.001) which was followed by Africa (b=1.35, *p* < 0.001). Conversely, the effect sizes for the East Asia and Pacific were relatively lower (b=1.07, *p* < 0.01).

None of the diagnostic tests showed a significant difference in effect sizes when compared with histopathology, as evident. With respect to specie, Plasmodium falciparum was the only specie with significantly higher effect size when compared to Plasmodium vivax in the meta regression analysis by specie.

## Quality assessment

All studies were included in the review after quality assessment. The JBI checklists for case–control, cohort, and cross-sectional studies were used according to the study designs (Table 3). Each study was scored out of the number of questions included in the checklist. The highest score was 10 for case–control studies, 11 for cohort studies, and 8 for cross-sectional studies.

**Table 3.** JBI appraisal checklist for included studies

Assessment of methodological quality											
JBI Appraisal Checklist for Case-Control Studies											
Study ID	Q1	Q2	Q3	Q4	Q5	Q6	Q7	Q8	Q9	Q10	Total
Babakhanyan 2016	Y	N	Y	Y	Y	N	N	Y	Y	Y	7/10
Mohammed 2013	Y	N	Y	Y	Y	N	N	Y	Y	Y	8/10
Monjol 2017	Y	N	Y	Y	Y	N	N	Y	Y	Y	7/10
Ndao 2009	Y	Y	Y	Y	Y	Y	Y	Y	Y	Y	10/10
Ofori 2018	Y	U	Y	Y	Y	N	N	Y	Y	Y	7/10
Singh 2012	Y	Y	Y	Y	Y	Y	Y	Y	Y	Y	10/10
Strand 2003	Y	N	Y	Y	Y	N	N	Y	Y	Y	7/10
Tahita 2013	Y	Y	Y	Y	Y	Y	Y	Y	Y	Y	10/10

JBI Appraisal Checklist for Cohort Studies												
Study ID	Q1	Q2	Q3	Q4	Q5	Q6	Q7	Q8	Q9	Q10	Q11	Total
Adegnika 2010	Y	Y	U	Y	Y	N	U	Y	Y	Y	Y	8/11
Akinnawo 2022	Y	Y	Y	Y	Y	N	Y	Y	Y	U	Y	9/11
Ayoola 2011	Y	Y	Y	Y	N	N	Y	Y	Y	Y	Y	9/11
Ayoola 2012	Y	Y	Y	N	N	N	Y	U	U	Y	Y	6/11
Bal 2023	Y	Y	Y	Y	Y	N	Y	Y	Y	U	Y	9/11
Bardají 2017	Y	Y	Y	Y	Y	N	Y	Y	Y	Y	Y	10/11
Boel 2010	Y	Y	Y	Y	N	N	Y	Y	Y	Y	Y	9/11
Boel 2012	Y	Y	Y	N	N	N	Y	Y	Y	Y	Y	8/11
Campos 2011	Y	Y	Y	N	N	N	Y	Y	Y	Y	Y	8/11
Carmona-Fonseca 2017	Y	Y	Y	Y	Y	N	Y	U	U	U	Y	7/11
Cohee 2014	Y	Y	Y	Y	Y	N	Y	Y	Y	Y	Y	10/11
Cot 2003	Y	Y	Y	Y	Y	N	Y	Y	Y	Y	Y	10/11
Cottrell 2006	Y	Y	Y	Y	Y	N	Y	Y	Y	Y	Y	10/11
Cottrell 2015	Y	Y	Y	Y	Y	N	Y	Y	Y	Y	Y	10/11
Dechavanne 2015a	Y	Y	Y	Y	Y	N	Y	Y	Y	Y	Y	10/11
Dechavanne 2015b	Y	Y	Y	N	N	N	Y	Y	Y	Y	Y	8/11
Elghazali 2003	Y	Y	Y	Y	Y	Y	Y	Y	Y	Y	Y	11/11
Fehintola 2012	Y	Y	Y	N	N	N	Y	Y	Y	N	Y	7/11
Fowkes 2018	Y	Y	Y	Y	Y	N	Y	Y	Y	Y	Y	10/11
Garrison 2022	Y	Y	Y	Y	Y	N	Y	Y	Y	Y	Y	10/11
Griffin 2012	Y	Y	Y	Y	Y	N	Y	Y	Y	Y	Y	10/11
Hounkonnou 2020	Y	Y	Y	Y	Y	N	Y	Y	Y	Y	Y	10/11
Hountohotegbe 2020	Y	Y	Y	Y	Y	N	Y	Y	Y	Y	Y	10/11
Huynh 2011	Y	Y	Y	Y	Y	N	Y	Y	Y	Y	Y	10/11
Kalilani 2010	Y	Y	Y	Y	Y	N	Y	Y	Y	Y	Y	10/11
Kalinjuma 2020	Y	Y	Y	Y	Y	N	Y	Y	Y	U	Y	9/11
Khan 2014	Y	Y	Y	Y	Y	N	Y	Y	Y	U	Y	9/11
Khattab 2013	Y	Y	Y	Y	Y	N	Y	U	U	U	Y	7/11
Koladjo 2022	Y	Y	Y	Y	Y	U	Y	Y	Y	Y	Y	10/11
Lamptey 2019	Y	Y	Y	Y	Y	N	Y	U	U	U	Y	7/11
Liu 2016b	Y	Y	Y	N	N	N	Y	Y	Y	U	Y	7/11
Lokossou 2013	Y	Y	Y	N	N	N	Y	U	U	U	Y	5/11
Manirakiza 2012	Y	Y	Y	Y	Y	N	Y	Y	Y	U	Y	9/11
Matambisso 2022	Y	Y	Y	Y	Y	N	Y	U	U	U	Y	7/11
Mayor 2018	Y	Y	Y	Y	Y	N	Y	Y	Y	U	Y	9/11
McClure 2014	Y	Y	Y	Y	Y	N	Y	U	U	Y	Y	8/11
McGready 2004	Y	Y	Y	N	N	N	Y	Y	Y	U	Y	7/11
McGready 2012	Y	Y	Y	N	N	N	Y	Y	Y	U	Y	7/11
McGregor 2017	Y	Y	Y	Y	Y	N	Y	U	U	U	Y	7/11
McLean 2017	Y	Y	Y	Y	Y	N	Y	Y	U	U	Y	8/11
McLean 2021	Y	Y	Y	Y	Y	N	Y	Y	Y	U	Y	9/11
Mosha 2014	Y	Y	Y	Y	Y	N	Y	U	U	U	Y	7/11
Msuya 2011	Y	Y	Y	N	N	N	Y	Y	Y	Y	Y	8/11
Mukhtar 2006	Y	Y	Y	N	N	N	Y	Y	Y	Y	Y	8/11
Nacher 2003	Y	Y	Y	Y	Y	N	Y	Y	Y	Y	Y	10/11
Natama 2018	Y	Y	Y	Y	Y	N	Y	Y	Y	Y	Y	10/11
Ndibazza 2013	Y	Y	Y	Y	Y	N	Y	Y	Y	Y	Y	10/11
Nkhoma 2012	Y	Y	Y	Y	Y	N	Y	Y	Y	U	Y	9/11
Oduwole 2011	Y	Y	Y	N	N	N	Y	U	U	U	Y	5/11
Ofori 2009	Y	Y	Y	N	N	N	Y	Y	Y	U	Y	7/11
Pincelli 2018	Y	Y	Y	Y	Y	N	Y	U	U	U	Y	7/11
Poespoprodjo 2011	Y	Y	Y	Y	Y	N	Y	Y	Y	U	Y	9/11
Poespoprodjo 2014	Y	Y	Y	Y	Y	N	Y	N	N	N	Y	7/11
Rijken 2012a	Y	Y	Y	Y	Y	N	Y	U	U	U	Y	7/11
Schmiegelow 2017	Y	Y	Y	Y	Y	N	Y	Y	Y	U	Y	9/11
Shannon 2016	Y	Y	Y	N	N	N	Y	Y	Y	U	Y	7/11
Singh 2001	Y	Y	Y	N	N	N	Y	Y	Y	U	Y	7/11
Singh 2014	Y	Y	Y	N	N	N	Y	Y	Y	Y	Y	8/11
Stanisic 2015	Y	Y	Y	Y	Y	N	Y	Y	Y	Y	Y	10/11
Stephens 2014	Y	Y	Y	N	N	N	Y	Y	Y	U	Y	7/11
Sylvester 2016	Y	Y	Y	Y	Y	N	Y	Y	Y	U	Y	9/11
Taylor 2017	Y	Y	Y	Y	Y	N	Y	Y	Y	U	Y	9/11
Teo 2014	Y	Y	Y	Y	Y	N	Y	Y	Y	U	Y	9/11
Tran 2020	Y	Y	Y	Y	Y	N	Y	Y	Y	Y	Y	10/11
Unger 2019	Y	Y	Y	Y	Y	N	Y	Y	Y	Y	Y	10/11
Unger 2022	Y	Y	Y	Y	Y	N	Y	U	U	U	Y	7/11
Vásquez 2018	Y	Y	Y	Y	Y	N	Y	U	U	U	Y	7/11
Verhoeff 2004	Y	Y	Y	Y	Y	N	Y	Y	Y	U	Y	9/11
Walther 2010	Y	Y	Y	Y	Y	N	Y	Y	Y	U	Y	9/11
Yovo 2022	Y	Y	Y	Y	Y	N	Y	Y	Y	Y	Y	10/11
Zhou 2002a	Y	Y	Y	Y	Y	N	Y	Y	Y	U	Y	10/11

JBI Appraisal Checklist for Analytical Cross-sectional Studies									
Study ID	Q1	Q2	Q3	Q4	Q5	Q6	Q7	Q8	Total
Abdelgadir 2012	Y	Y	Y	Y	N	N	Y	Y	6/8
Abdelrahim 2009	Y	Y	Y	Y	N	N	Y	Y	6/8
Adam 2005	N	Y	Y	Y	Y	Y	Y	Y	7/8
Adam 2007	Y	Y	Y	Y	N	N	Y	Y	6/8
Adam 2008	Y	Y	Y	Y	N	N	Y	Y	6/8
Adam 2009	Y	Y	Y	Y	N	N	Y	Y	6/8
Adam 2012	N	Y	Y	Y	N	N	Y	Y	5/8
Adam 2017	Y	Y	Y	Y	N	N	Y	Y	6/8
Adegnika 2006	Y	Y	Y	Y	Y	Y	Y	Y	8/8
Afutu 2021	Y	Y	Y	Y	N	N	Y	Y	6/8
Agu 2013	N	Y	Y	Y	Y	Y	Y	Y	7/8
Agudelo 2013	Y	Y	Y	Y	N	N	Y	Y	6/8
Aguilar 2012	N	Y	Y	Y	N	N	Y	Y	5/8
Aguzie 2017	N	Y	Y	Y	N	N	Y	Y	5/8
Ahadzie-Soglie 2022	Y	Y	Y	Y	Y	Y	Y	Y	8/8
Ahenkorah 2020	Y	Y	Y	Y	Y	Y	Y	Y	8/8
Ahmed 2014	N	Y	Y	Y	Y	Y	Y	Y	7/8
Ahmed 2015	U	Y	Y	Y	N	N	Y	Y	5/8
Aliyu 2017	Y	Y	Y	Y	N	N	Y	Y	5/8
Almaw 2022	Y	Y	Y	Y	Y	Y	Y	Y	8/8
Anabire 2019a	N	Y	Y	Y	Y	Y	Y	Y	7/8
Anabire 2019b	Y	Y	Y	Y	N	N	Y	Y	6/8
Anabire 2023	Y	Y	Y	Y	N	N	Y	Y	6/8
Anchang-Kimbi 2015	U	Y	Y	Y	Y	Y	Y	Y	7/8
Anchang-Kimbi 2017	U	Y	Y	Y	N	N	Y	Y	7/8
Anchang-Kimbi 2020	Y	Y	Y	Y	Y	Y	Y	Y	8/8
Appleyard 2008	N	Y	Y	Y	N	N	Y	Y	5/8
Ataíde 2010	Y	Y	Y	Y	Y	Y	Y	Y	8/8
Ataíde 2015	Y	Y	Y	Y	Y	Y	Y	Y	8/8
Atakorah 2022	Y	Y	Y	Y	N	N	Y	Y	6/8
Avery 2012	Y	Y	Y	Y	Y	Y	Y	Y	8/8
Ayoya 2006	Y	Y	Y	Y	Y	Y	Y	Y	8/8
Balcha 2023	Y	Y	Y	Y	N	N	Y	Y	6/8
Bassey 2015	Y	Y	Y	Y	N	N	Y	Y	6/8
Bassiouny 2005	N	Y	Y	Y	N	N	Y	Y	5/8
Bedu-Addo 2014	N	Y	Y	Y	Y	Y	Y	Y	7/8
Benet 2006	U	Y	Y	Y	Y	Y	Y	Y	7/8
Biteghe-Bi-Essone 2022	Y	Y	Y	Y	Y	Y	Y	Y	8/8
Blay 2015	U	Y	Y	Y	Y	Y	Y	Y	7/8
Bouyou-Akotet 2003	U	Y	Y	Y	Y	Y	Y	Y	7/8
Bouyou-Akotet 2004	N	Y	Y	Y	N	N	Y	Y	5/8
Bouyou-Akotet 2016	Y	Y	Y	Y	Y	Y	Y	Y	8/8
Bracho 2022	N	U	Y	U	N	N	Y	Y	3/8
Braun 2015	N	Y	Y	Y	N	N	Y	Y	5/8
Briand 2016	Y	Y	Y	Y	Y	Y	Y	Y	8/8
Brutus 2013	Y	Y	Y	Y	Y	Y	Y	Y	8/8
Campos 2012	Y	Y	Y	Y	N	N	Y	Y	6/8
Cardona-Arias 2022	Y	Y	Y	Y	Y	Y	Y	Y	8/8
Cardona-Arias 2023	Y	Y	Y	Y	N	N	Y	Y	6/8
Carrara 2013	N	Y	Y	Y	Y	Y	Y	Y	7/8
Chaponda 2015	Y	Y	Y	Y	Y	Y	Y	Y	8/8
Cisse 2014	U	Y	Y	Y	Y	Y	Y	Y	7/8
Corrêa 2017	Y	Y	Y	Y	N	N	Y	Y	6/8
Djontu 2016	Y	Y	Y	Y	N	N	Y	Y	6/8
Doritchamou 2013	N	U	Y	Y	N	N	Y	Y	4/8
Dosoo 2020	Y	Y	Y	Y	Y	Y	Y	Y	8/8
Douamba 2012	Y	Y	Y	Y	N	N	Y	Y	6/8
Ebong 2022	Y	Y	Y	Y	N	N	Y	Y	6/8
Efunshile 2011	Y	Y	Y	Y	N	N	Y	Y	6/8
Elbashir 2011	Y	Y	Y	Y	N	N	Y	Y	6/8
Enato 2009	U	Y	Y	Y	N	N	Y	Y	5/8
Esu 2018	Y	Y	Y	Y	N	N	Y	Y	6/8
Fagbemi 2020	Y	Y	Y	Y	N	N	Y	Y	6/8
Fairley 2013	Y	Y	Y	Y	Y	Y	Y	Y	8/8
Feleke 2020	Y	Y	Y	Y	Y	Y	Y	Y	8/8
Francine 2016	Y	Y	Y	Y	Y	Y	Y	Y	8/8
Fusai 2000	N	N	Y	Y	N	N	Y	Y	4/8
Godwin 2022	Y	Y	Y	Y	Y	Y	Y	Y	8/8
Gontie 2020	Y	Y	Y	Y	Y	Y	Y	Y	8/8
Gutman 2015	Y	Y	Y	Y	Y	Y	Y	Y	8/8
Hamann 2010	Y	Y	Y	Y	Y	Y	Y	Y	8/8
Hamer 2009	Y	Y	Y	Y	Y	Y	Y	Y	8/8
Helegbe 2018	Y	Y	Y	Y	Y	Y	Y	Y	8/8
Ikegbunam 2019	Y	Y	Y	Y	N	N	Y	Y	6/8
Ikegbunam 2022	Y	Y	Y	Y	N	N	Y	Y	6/8
Iwalokun 2015	Y	Y	Y	Y	N	N	Y	Y	6/8
Jäckle 2013	Y	Y	Y	Y	Y	Y	Y	Y	8/8
Jaén-Sánchez 2023a	Y	Y	Y	Y	Y	Y	Y	Y	8/8
Jaén-Sánchez 2023b	Y	Y	Y	Y	N	N	Y	Y	6/8
Jeza 2022	Y	Y	Y	Y	Y	Y	Y	Y	8/8
Kabanywanyi 2008	Y	Y	Y	Y	N	N	Y	Y	6/8
Kagu 2007	Y	Y	Y	Y	N	N	Y	Y	6/8
Kasumba 2000	U	Y	Y	Y	N	N	Y	Y	5/8
Kattenberg 2012	U	Y	Y	Y	N	N	Y	Y	5/8
Kayiba 2021	Y	Y	Y	Y	N	N	Y	Y	6/8
King 2021	Y	Y	Y	Y	N	N	Y	Y	6/8
Koukouikila-Koussounda 2015	Y	Y	Y	Y	N	N	Y	Y	6/8
Kurth 2010	Y	Y	Y	Y	Y	Y	Y	Y	8/8
Lingani 2022	Y	Y	Y	Y	Y	Y	Y	Y	8/8
Liu 2016a	N	U	Y	Y	N	N	Y	Y	4/8
Maïga-Ascofaré 2015	N	Y	Y	Y	N	N	Y	Y	5/8
Mama 2022	Y	Y	Y	Y	Y	Y	Y	Y	8/8
Mankhambo 2002	Y	Y	Y	Y	Y	Y	Y	Y	8/8
Martínez-Pérez 2018	Y	Y	Y	Y	N	N	Y	Y	6/8
Matangila 2014	Y	Y	Y	Y	Y	Y	Y	Y	8/8
Mayengue 2004	Y	Y	Y	Y	Y	Y	Y	Y	8/8
Mayor 2009	Y	Y	Y	Y	N	N	Y	Y	6/8
Mbacham 2023	Y	Y	Y	Y	Y	Y	Y	Y	8/8
Mbonye 2013	Y	Y	Y	Y	N	N	Y	Y	6/8
Mbouamboua 2019	Y	Y	Y	Y	Y	Y	Y	Y	8/8
Megnekou 2015	Y	Y	Y	Y	Y	Y	Y	Y	8/8
Megnekou 2018	Y	Y	Y	Y	Y	Y	Y	Y	8/8
Minang 2004	Y	Y	Y	Y	U	U	Y	Y	6/8
Mlugu 2020	Y	Y	Y	Y	Y	Y	Y	Y	8/8
Mockenhaupt 2001	Y	Y	Y	Y	N	N	Y	Y	6/8
Mockenhaupt 2002	Y	Y	Y	Y	N	N	Y	U	5/8
Mockenhaupt 2003	Y	Y	Y	Y	Y	Y	Y	Y	8/8
Mockenhaupt 2006	Y	Y	Y	Y	Y	Y	Y	Y	8/8
Mockenhaupt 2008	Y	Y	Y	Y	N	N	Y	Y	6/8
Moore 2016	Y	Y	Y	Y	Y	Y	Y	Y	8/8
Moore 2017	Y	Y	Y	Y	Y	Y	Y	Y	8/8
Muhangi 2007	Y	Y	Y	Y	Y	Y	Y	Y	8/8
Mwin 2021	Y	Y	Y	Y	Y	Y	Y	Y	8/8
Nega 2015	Y	Y	Y	Y	Y	Y	Y	Y	8/8
Nekaka 2020	Y	Y	Y	Y	Y	Y	Y	Y	8/8
Newman 2003	Y	Y	Y	Y	N	N	Y	Y	6/8
Niang 2008	Y	Y	Y	Y	Y	Y	Y	Y	8/8
Nlinwe 2022	Y	Y	Y	Y	N	N	Y	Y	6/8
Ntoumi 2013	Y	Y	Y	Y	U	U	Y	Y	6/8
Nwaefuna 2015	Y	Y	Y	Y	Y	Y	Y	Y	8/8
Nyamu 2020	Y	Y	Y	Y	Y	Y	Y	Y	8/8
Obiri 2020	Y	Y	Y	Y	Y	Y	Y	Y	8/8
Ogbodo 2009	U	Y	Y	Y	N	U	Y	Y	5/8
Ojurongbe 2011	Y	Y	Y	Y	N	N	Y	Y	6/8
Ojurongbe 2018a	Y	Y	Y	Y	N	N	Y	Y	6/8
Ojurongbe 2018b	Y	Y	Y	Y	N	N	Y	Y	6/8
Okafor 2006	Y	Y	Y	Y	N	N	Y	Y	6/8
Okoko 2001	Y	Y	Y	Y	N	N	Y	Y	6/8
Omer 2011	N	Y	Y	Y	Y	Y	Y	Y	7/8
Omer 2017	Y	Y	Y	Y	N	N	Y	Y	6/8
Oraneli 2013	U	Y	Y	Y	N	N	Y	Y	5/8
Orish 2012	Y	Y	Y	Y	Y	Y	Y	Y	8/8
Osarfo 2017	Y	Y	Y	Y	Y	Y	Y	Y	8/8
Ouédraogo 2019	N	Y	Y	Y	N	N	Y	Y	5/8
Oyeyemi 2016	Y	Y	Y	Y	N	N	Y	Y	6/8
Patel 2016	Y	Y	Y	Y	Y	Y	Y	Y	8/8
Perrault 2009	Y	Y	Y	Y	Y	Y	Y	Y	8/8
Plotkin 2014	Y	Y	Y	Y	N	U	Y	Y	6/8
Poespoprodjo 2008	N	Y	Y	Y	Y	Y	Y	Y	7/8
Pujol 2023	Y	Y	Y	Y	N	N	Y	Y	6/8
Quakyi 2019	U	Y	Y	Y	Y	Y	Y	Y	7/8
Rijken 2012b	Y	Y	Y	Y	Y	Y	Y	Y	8/8
Rogerson 2000a	N	Y	Y	Y	N	N	Y	Y	5/8
Rogerson 2000b	Y	Y	Y	Y	Y	Y	Y	Y	8/8
Rogerson 2003a	U	U	Y	Y	N	N	Y	Y	4/8
Rogerson 2003b	U	U	Y	Y	Y	Y	Y	Y	6/8
Romagosa 2004	Y	Y	Y	Y	N	N	Y	Y	6/8
Ruh 2018	N	Y	Y	Y	N	N	Y	U	4/8
Salifu 2016	Y	Y	Y	Y	N	N	Y	Y	6/8
Salih 2011	Y	U	Y	Y	N	N	Y	Y	5/8
Samuels 2022	Y	Y	Y	Y	N	N	Y	Y	6/8
Singh 2020	Y	Y	Y	Y	Y	Y	Y	Y	8/8
Sirima 2003	Y	Y	Y	Y	Y	Y	Y	Y	8/8
Sohail 2015	Y	Y	Y	Y	Y	Y	Y	Y	8/8
Soulard 2011	Y	Y	Y	Y	Y	Y	Y	Y	8/8
Subussa 2021	Y	Y	Y	Y	N	N	Y	Y	6/8
Tako 2005	Y	Y	Y	Y	Y	Y	Y	Y	8/8
Toure 2014	Y	Y	Y	Y	Y	Y	Y	Y	8/8
Ugwu 2014	Y	Y	Y	Y	N	N	Y	Y	6/8
Ukaga 2007	Y	Y	Y	Y	N	N	Y	Y	6/8
Uneke 2007	Y	Y	Y	Y	N	N	Y	Y	6/8
Valente 2011	Y	Y	Y	Y	Y	Y	Y	Y	8/8
vanEijk 2001	Y	Y	Y	Y	Y	Y	Y	Y	8/8
vanEijk 2009	Y	Y	Y	Y	Y	Y	Y	Y	8/8
VanGeertruyden 2005	Y	Y	Y	Y	Y	Y	Y	Y	8/8
vanLenthe 2019	Y	Y	Y	Y	N	N	Y	Y	6/8
vanLoon 2019	Y	Y	Y	Y	Y	Y	Y	Y	8/8
Vásquez 2020a	Y	Y	Y	Y	N	N	Y	Y	6/8
Williams 2016	Y	Y	Y	Y	Y	Y	Y	Y	8/8
Woodburn 2009	Y	Y	Y	Y	Y	Y	Y	Y	8/8
Wumba 2015	Y	Y	Y	Y	Y	Y	Y	Y	8/8
Yatich 2010	Y	Y	Y	Y	N	N	Y	Y	6/8
Yeboah 2016	Y	Y	Y	Y	N	N	Y	Y	6/8
Zablon 2015	Y	Y	Y	Y	N	N	Y	Y	6/8

Abbreviations: Y, Yes; N, No; U, Unclear; N/A, Not Applicable.

Out of the 8 case–control studies, three studies scored 10/10, one study scored 8/10, and four studies scored 7/10. Of the 71 cohort studies, one study scored 11/11, twenty-two studies scored 10/11, seventeen studies scored 8/11, nineteen studies scored 7/11, one study scored 6/11, and two studies scored 5/11. Of the 174 cross-sectional studies, seventy-one studies scored 8/8, fifteen studies scored 7/8, sixty-three studies scored 6/8, nineteen studies scored 5/8, five studies scored 4/8, and one study scored 3/8.

The most common problems that came across overall were the identification of confounding factors and strategies to deal with confounding factors were not mentioned clearly. In the cohort studies, the most common problem was that the subjects were not free of the outcome at the start of the study and strategies to deal with incomplete follow-up were not clearly mentioned.

## Discussion

Malaria in pregnancy is a cause of extensive morbidity and mortality globally, both among infectious diseases and overall. While numerous studies have estimated the rate of infection in different regions, this meta-analysis synthesizes an immense volume of data to describe the overall prevalence and distribution of the disease. The findings of our study highlight that prevalence of malaria varies geographically, temporally, and species specifically. Amongst the many virulent species, *Plasmodium falciparum* has been the cause of highest incidence of infection. Similarly, African region has shown highest regional prevalence amongst the other regions. In addition, prevalence was higher during the antenatal visits as opposed to at delivery.

In addition, we have secondarily analysed and demonstrated that several morbid disease states and outcomes, such as anaemia, low birthweight, preterm birth, and stillbirth, may be significantly associated with malaria during pregnancy. These detrimental factors to the well-being and survival of mothers and their infants may influence maldevelopment and poor health in individuals throughout the life-course if left unaddressed.

As estimated by our study, Africa presents with the highest burden of malaria in pregnancy. This is in line with studies conducted earlier in the region and the report presented by the World Health Organization [2, 18–20]. This may be due to malarial endemicity of the region as it is considered as the most tropical continent, coupled with higher transmissibility of the infection. This endemicity is the product of a complex interplay of environmental, biological, and socio-economic factors. Tropical climates with appropriate temperature, humidity, and rainfall conditions encourage endemicity of the disease as they are conducive to the reproduction of the parasite within the anopheles’ mosquito, which is itself native to these environments [17].

However, this natural localization of malaria is compounded by a lack of robust and resilient health systems in many of the affected countries, where poverty, conflict, and natural disasters often further limit the impact of concerted public health efforts to tackle the disease [14, 15]. To counter, preventive measures and immunogenicity of the population play a very significant role in combatting the pathogenesis of disease in any geographical region. Thus, the prevalence has reduced within Africa but is still the highest amongst other regions [21]. Even though the studies of Africa have shown a significant reduction in the prevalence of malaria, it is worth noting that these measures have not accounted for all the countries in the region, hence limiting its generalizability [11].

In this study, we also observed that Plasmodium falciparum was responsible for the pathogenicity of the majority of infections. Several systematic reviews have confirmed that P. falciparum is the highest inhabited organism in pregnancy to cause the infection [7, 22]. Our study’s findings of a disproportionately high prevalence of this organism of malaria underscore the importance of taking strong measures to prevent and manage the disease, especially among pregnant women. While the WHO malaria 2016 report found that over 99% of malaria cases were attributable to P. falciparum, our analysis found a smaller proportion of P. falciparum-causing illnesses [23]). Extreme seasonal, interannual, and geographical fluctuation may be responsible for these shifts. Possible causes include dissimilarities in development and housing patterns, population migration, as well as climatic (temperature, precipitation, and relative humidity) factors.

The study assessment also revealed that malaria-positive women were more prone to encounter anaemia. Several meta-analyses support our findings as the overall odds of malaria of anaemia are higher amongst pregnant women with malaria [24]. According to a review, malaria is responsible for an estimated 26% of the severe anaemia experienced by pregnant women of all gravities (population attributable fraction) [7]. Anaemia is strongly linked to malaria, although the underlying pathophysiology is poorly understood. Nonetheless, illness-related inadequate food intake, haemolysis, and a lack of micronutrients are all viable justifications for anaemia and malaria.

Association of low birthweight with the presence of maternal malaria was amongst the deductions from our study. This is validated by other reviews conducted that suggest the same statistically significant association between malaria in pregnancy and low birthweight of the baby [25]. Around 19% of LBWs and 6% of LBW-related infant fatalities are attributed to malaria in regions where the disease is endemic. According to these estimates, over 100,000 infants die each year in parts of Africa where malaria is common because to LBW [26].

Augmenting with the findings of our study related to preterm babies and malaria exposure, several reviews have reported malaria to be the primary infection in pregnancy that can be associated with the PTB [27]. Moreover, PTB seasonality patterns were also observed in some studies to be paralleling those of malaria infection, with its peak occurring with periods of high malaria infection [28].

Our study also revealed that proportions of stillbirths were higher with women with malaria in pregnancy. This has been validated by other reviews conducted earlier that have reported a widespread effect of malaria and risk of stillbirths [10, 29]. Amongst the major modifiable risk factors of stillbirths, risk attributed to malaria is approximately 8% which can be prevented if exposure minimized [30].

Amongst the major strengths of the review, the inclusion of 253 studies determining the burden of malaria in pregnancy creates a substantial mark. It gives us a holistic global standpoint of prevalence of the disease and its association with adverse pregnancy outcomes on both the maternal and neonatal health. To further strengthen the robustness of the review, sensitivity analyses were performed which refined the effect sizes of the meta-analyses eliminating the influential studies. In addition, assessment of publication bias was also undertaken to identify the presence of biases via relevant plots.

The limitations of the review include the non-uniformity of diagnostic test used. Multiple approaches, varying in sensitivity and specificity, were used to detect malaria during pregnancy. Not all studies utilize PCR for logistical reasons, and microscopy and rapid diagnostic tests are vulnerable to errors depending on reagents, personnel, mutant strains, and other factors. It is also pertinent to note that we lacked access to individual patient data from the studies that yielded adjusted estimates; thus, we were unable to account for this variation. Since the factors adjusted were not uniform in all studies, dichotomous data were preferred as a measure of reported and studies that failed to report dichotomous data were excluded. Further, confounding was also not taken into consideration when deducing associations with adverse outcomes and we also could not conduct the association analysis by strain due to paucity and diversity of data, which did not allow us to do a sub-group analysis.

## Conclusion

Despite significant work being done to control the spread of the disease, the burden of malaria persists. A substantial impact of unfavourable pregnancy outcome also adds up to the seriousness of the issue and requires urgent attention and concern. Large-scale interventional studies are the need of the time to address this public health issue along with global level policy formulations to target the vulnerable populations living with such elevated burden of disease.

## Supporting information

Das et al. supplementary material Das et al. supplementary material

## Data Availability

Data are available upon reasonable request. All data relevant to the study is included in the article.

## Supplementary material

For supplementary material accompanying this paper visit http://doi.org/10.1017/S0950268824000177. click here to view supplementary material
