# Supplementary figures and images for: Malaria in pregnancy: Meta-analyses of prevalence and associated complications
Source: Epidemiol Infect. 2024 Feb 13;152:e39. doi: 10.1017/S0950268824000177 (PMC10945947; doi:10.1017/S0950268824000177)

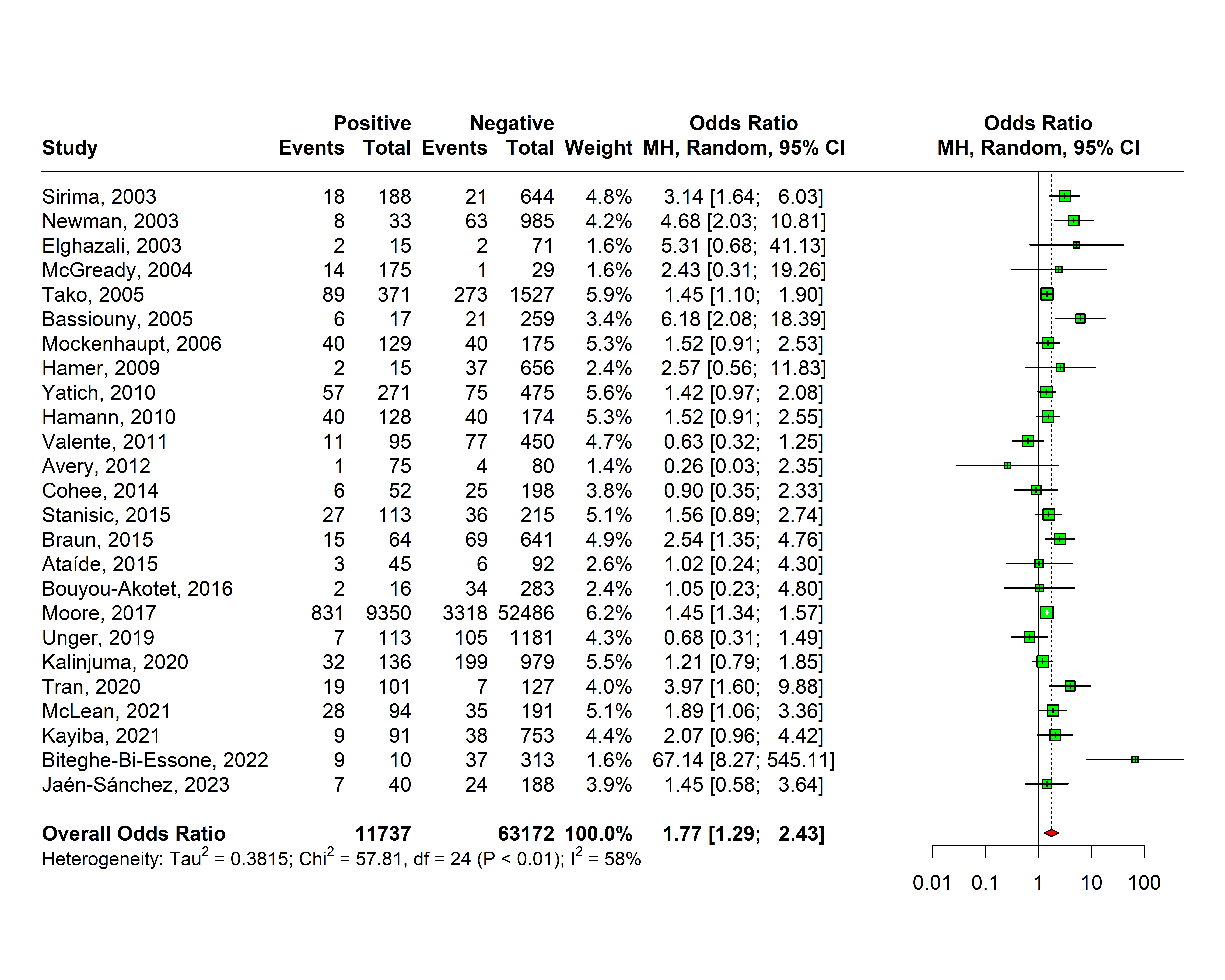

Supplement: Das et al. supplementary material [file S0950268824000177sup001.zip › S0950268824000177sup001.png]

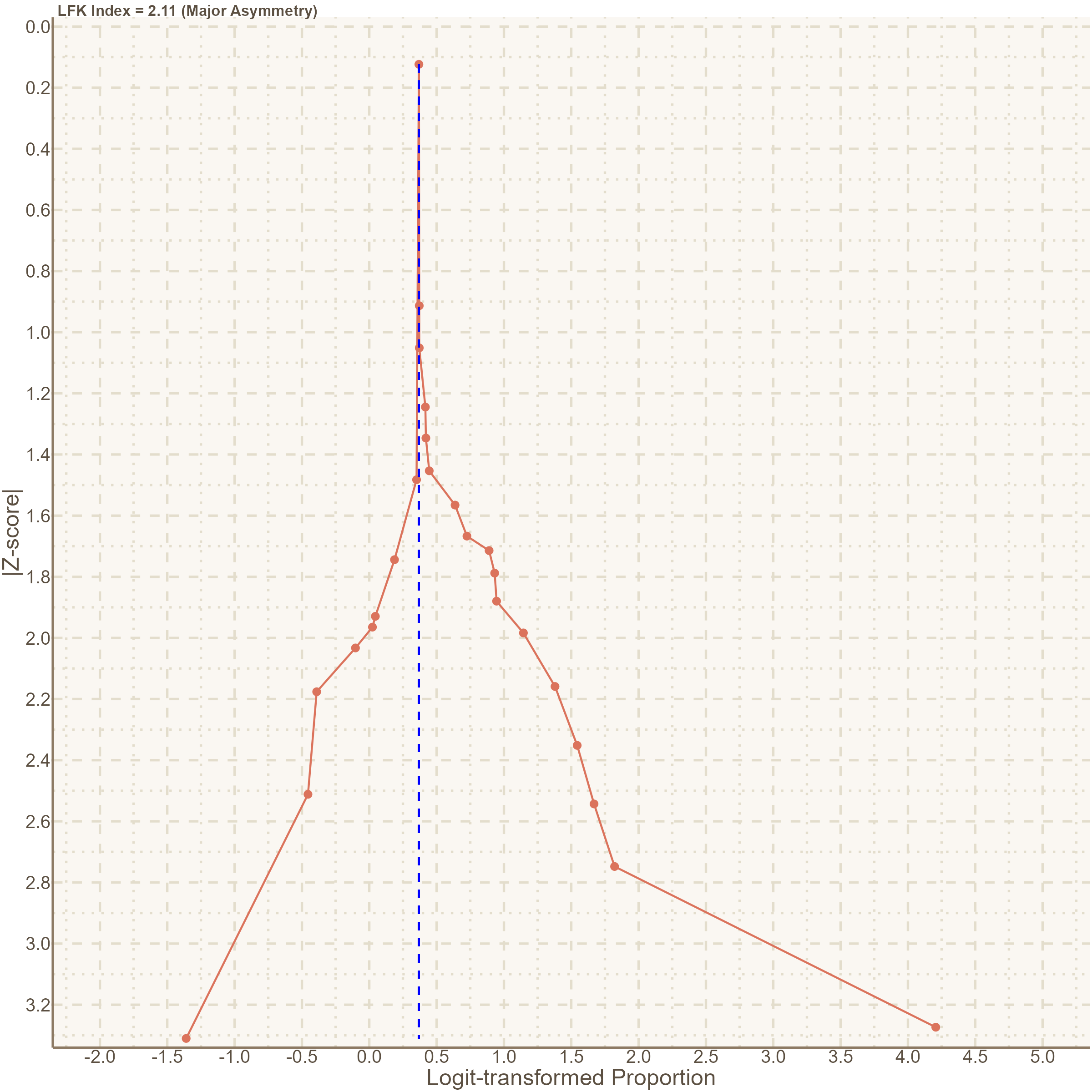

Supplement: Das et al. supplementary material [file S0950268824000177sup001.zip › S0950268824000177sup002.tiff]

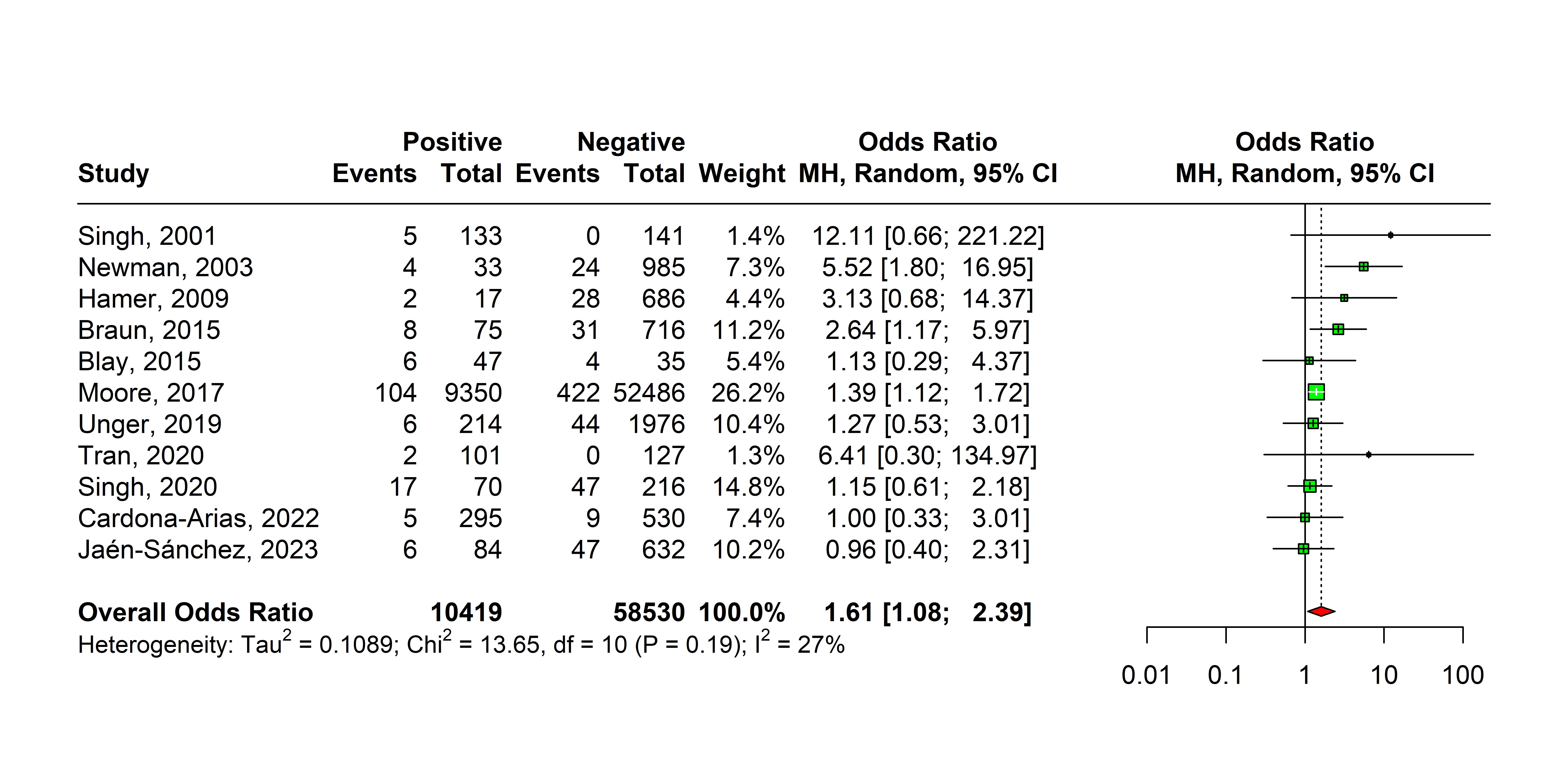

Supplement: Das et al. supplementary material [file S0950268824000177sup001.zip › S0950268824000177sup003.png]

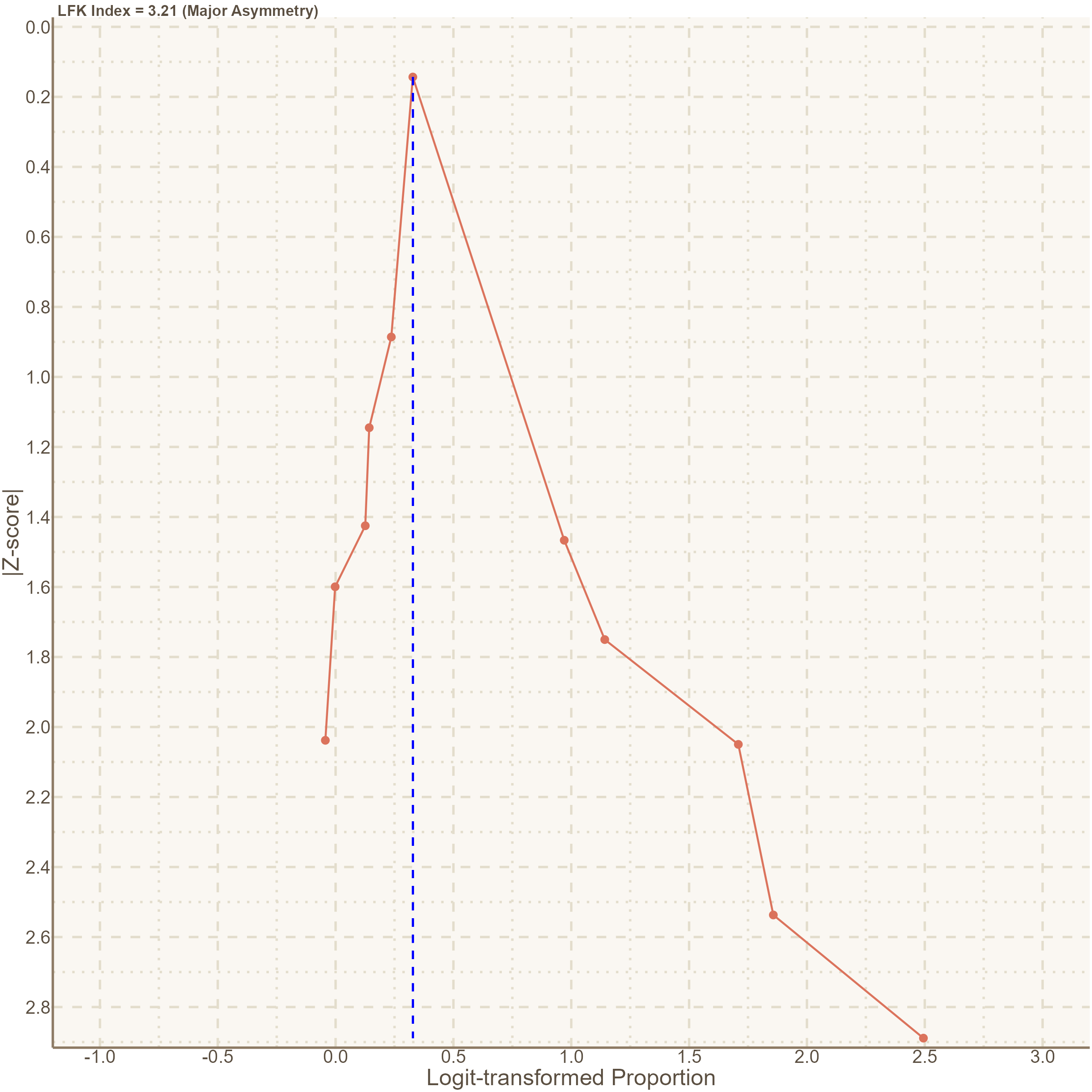

Supplement: Das et al. supplementary material [file S0950268824000177sup001.zip › S0950268824000177sup004.png]

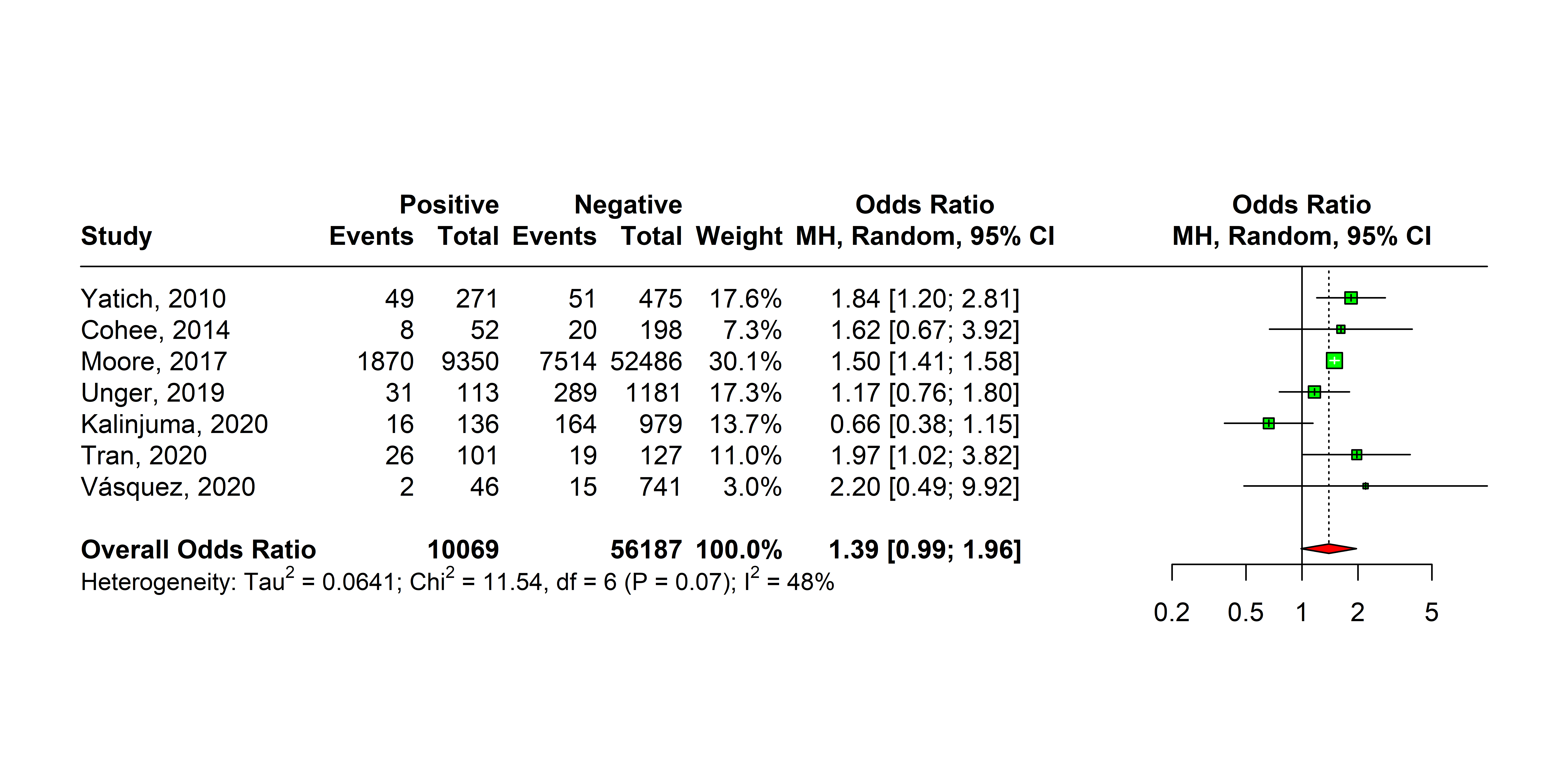

Supplement: Das et al. supplementary material [file S0950268824000177sup001.zip › S0950268824000177sup005.png]

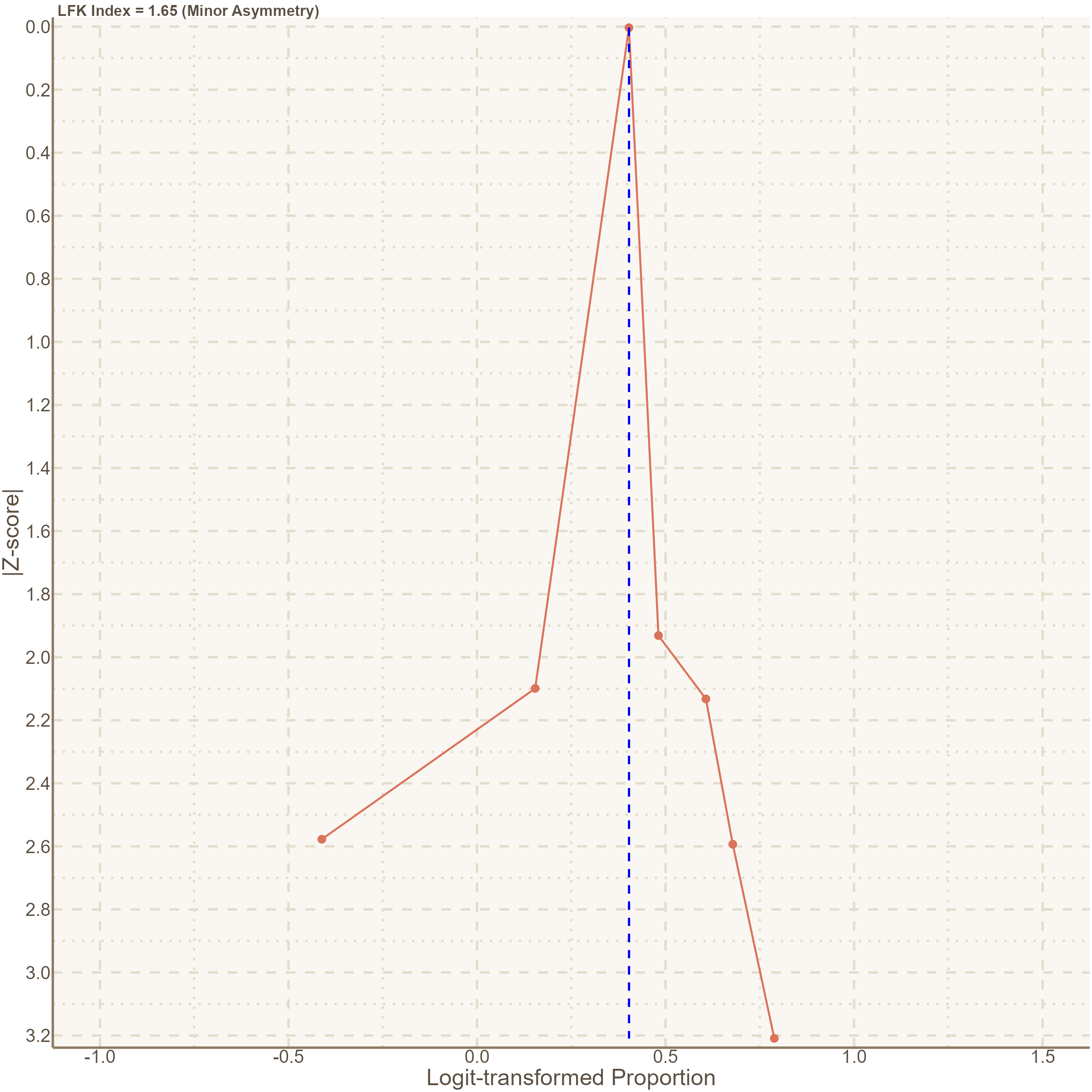

Supplement: Das et al. supplementary material [file S0950268824000177sup001.zip › S0950268824000177sup006.png]

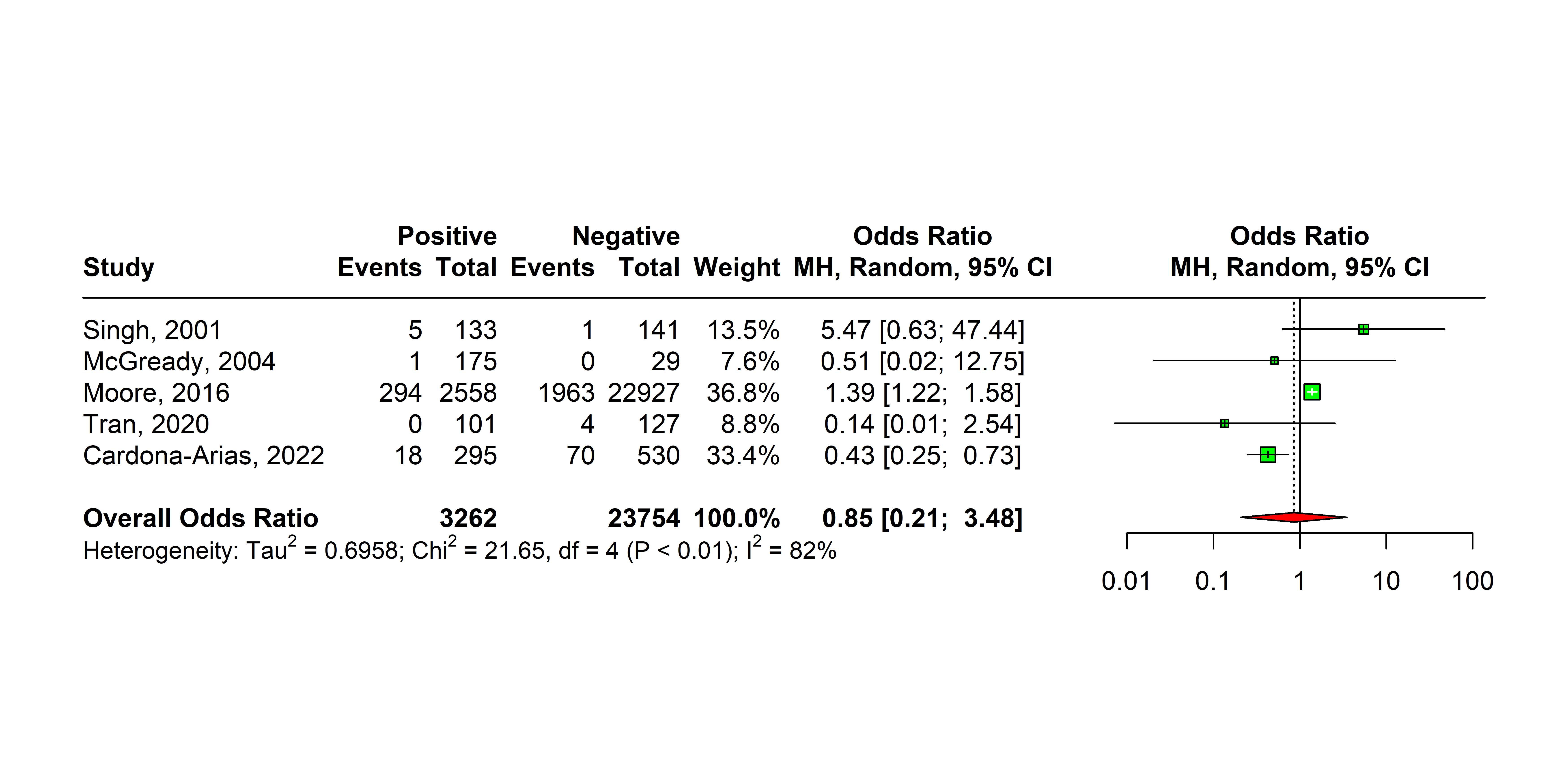

Supplement: Das et al. supplementary material [file S0950268824000177sup001.zip › S0950268824000177sup007.png]

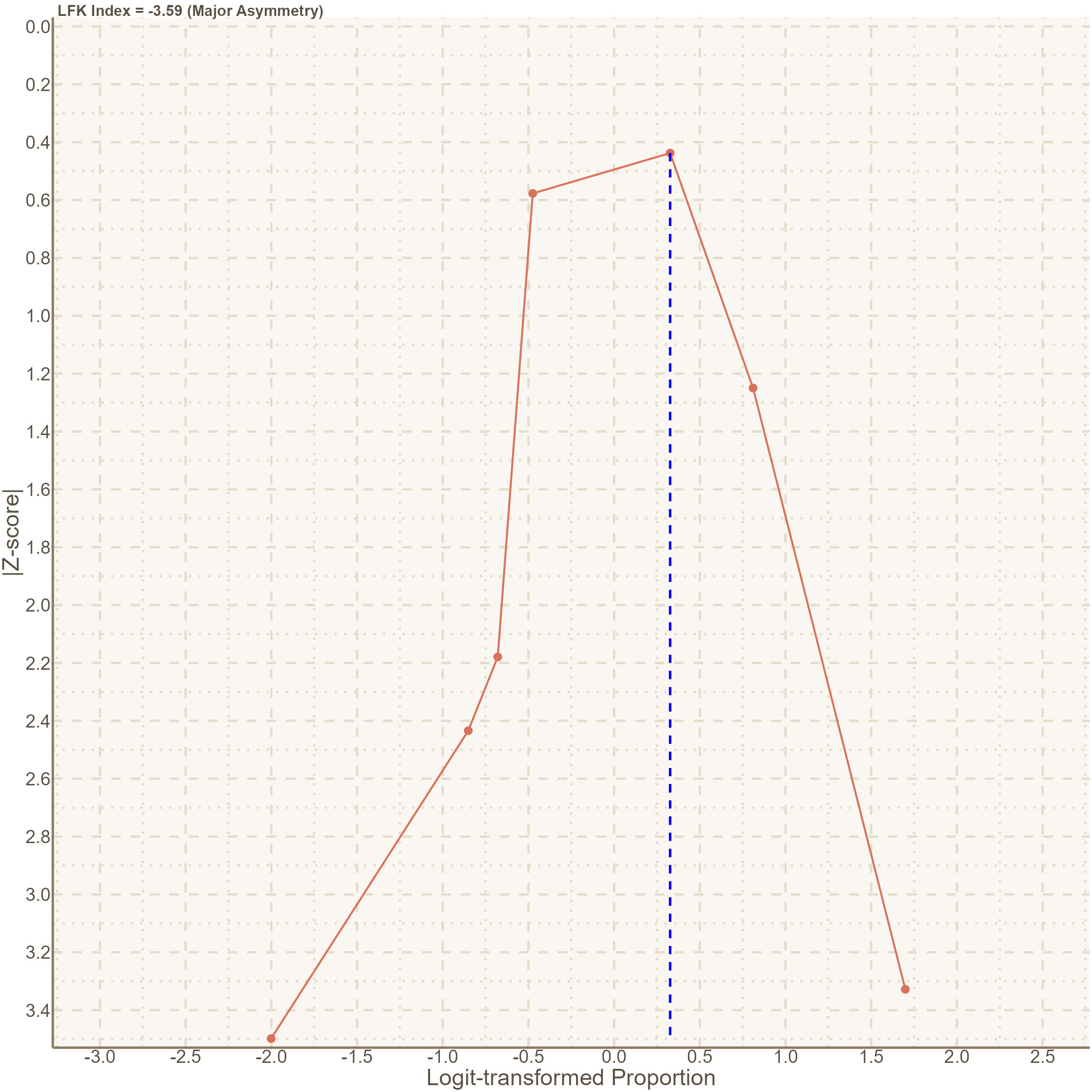

Supplement: Das et al. supplementary material [file S0950268824000177sup001.zip › S0950268824000177sup008.png]

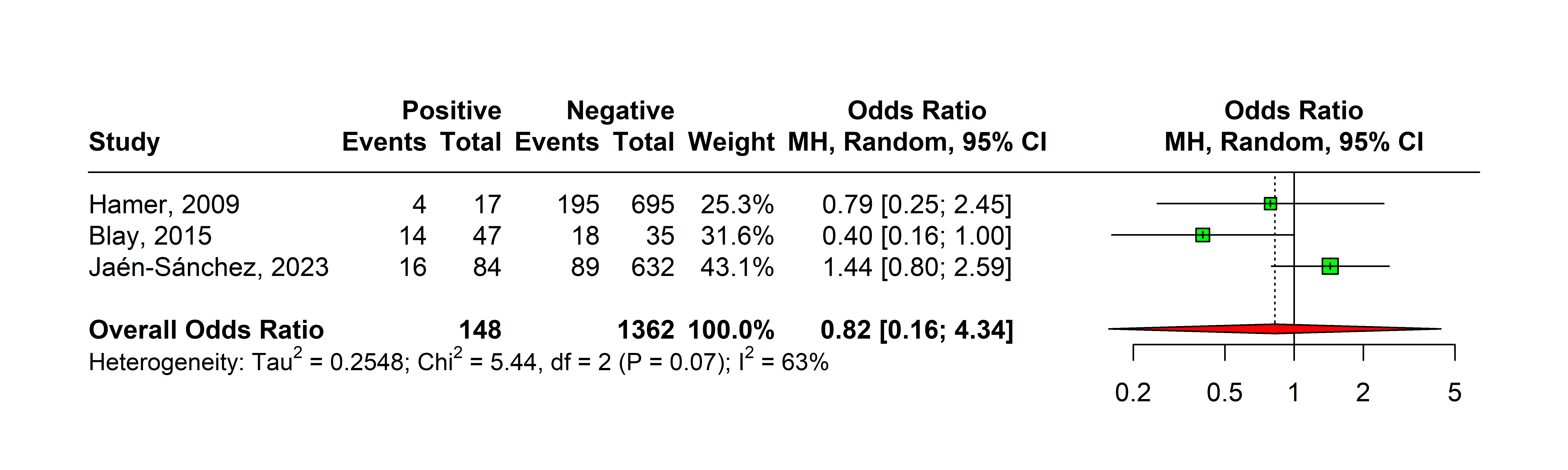

Supplement: Das et al. supplementary material [file S0950268824000177sup001.zip › S0950268824000177sup009.png]

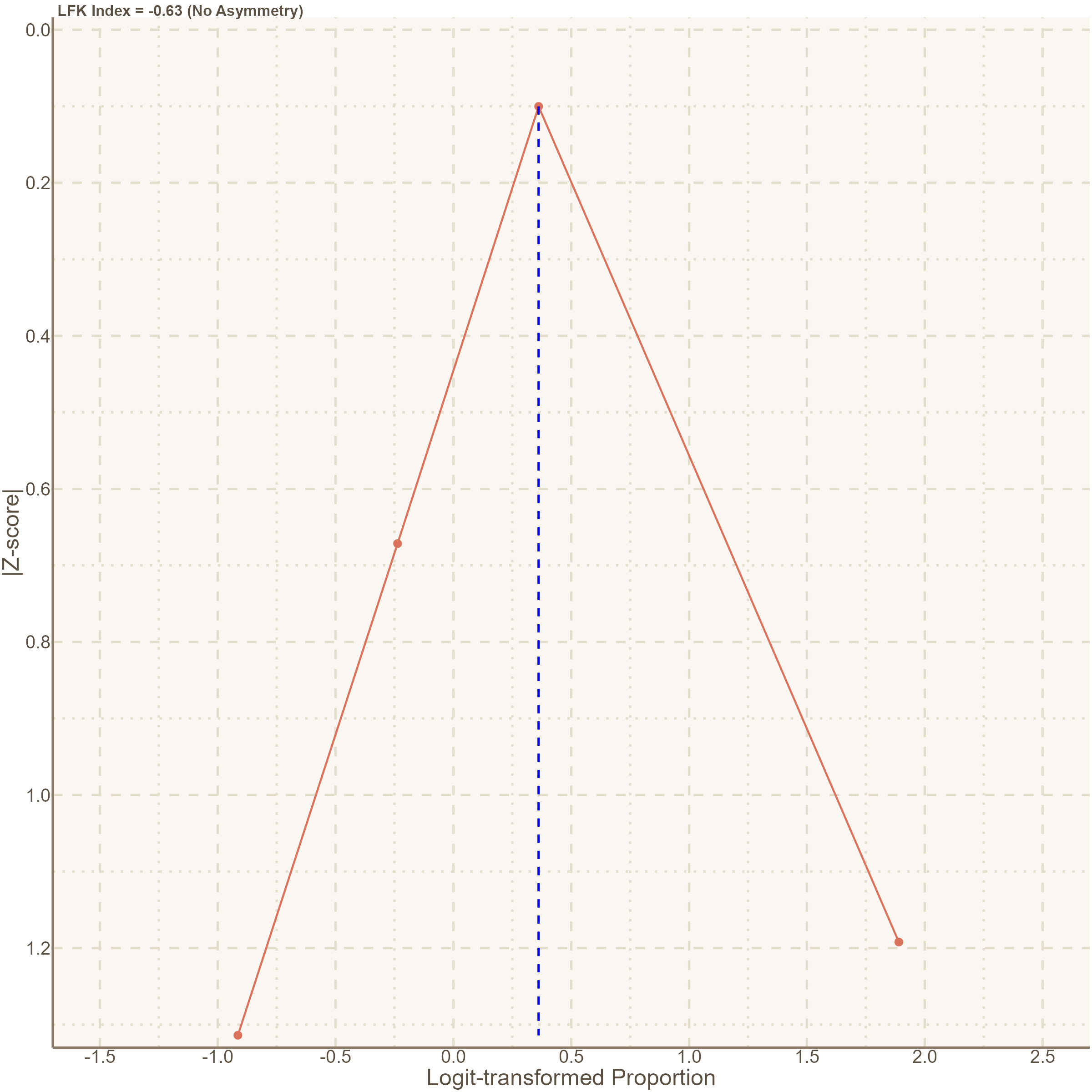

Supplement: Das et al. supplementary material [file S0950268824000177sup001.zip › S0950268824000177sup010.png]

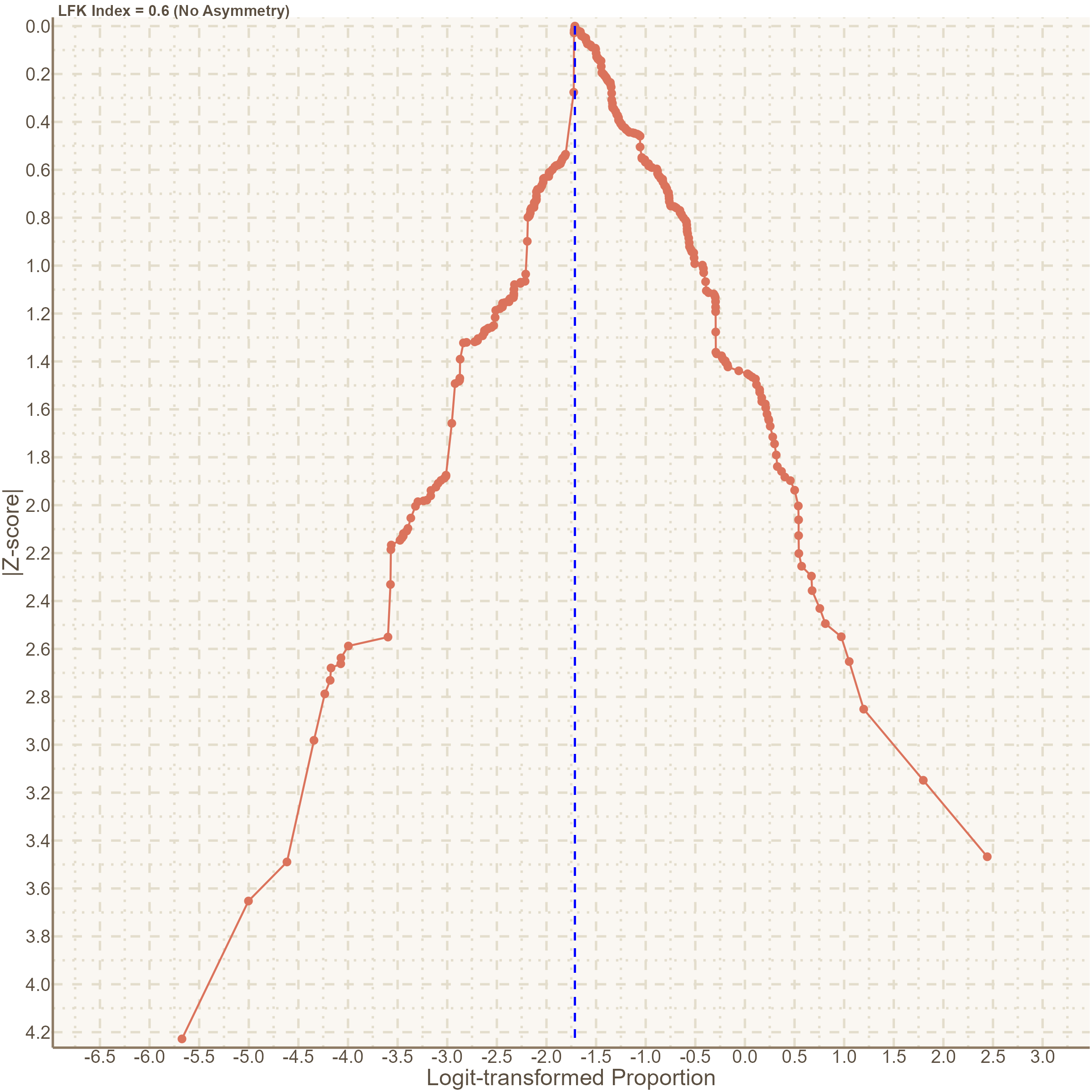

Supplement: Das et al. supplementary material [file S0950268824000177sup001.zip › S0950268824000177sup012.png]

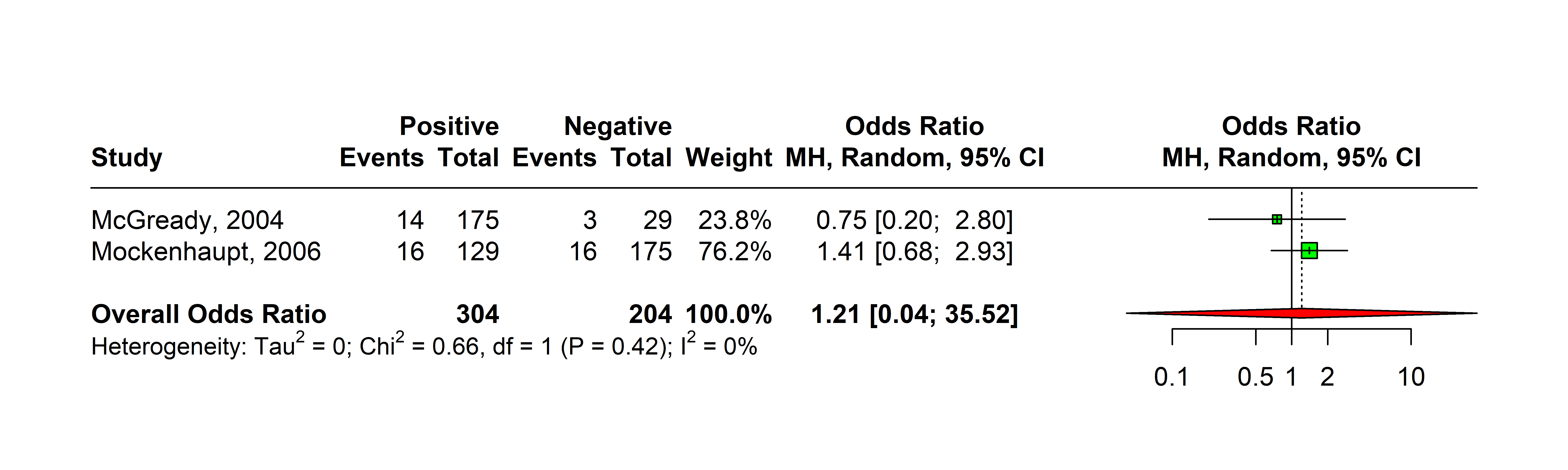

Supplement: Das et al. supplementary material [file S0950268824000177sup001.zip › S0950268824000177sup013.png]

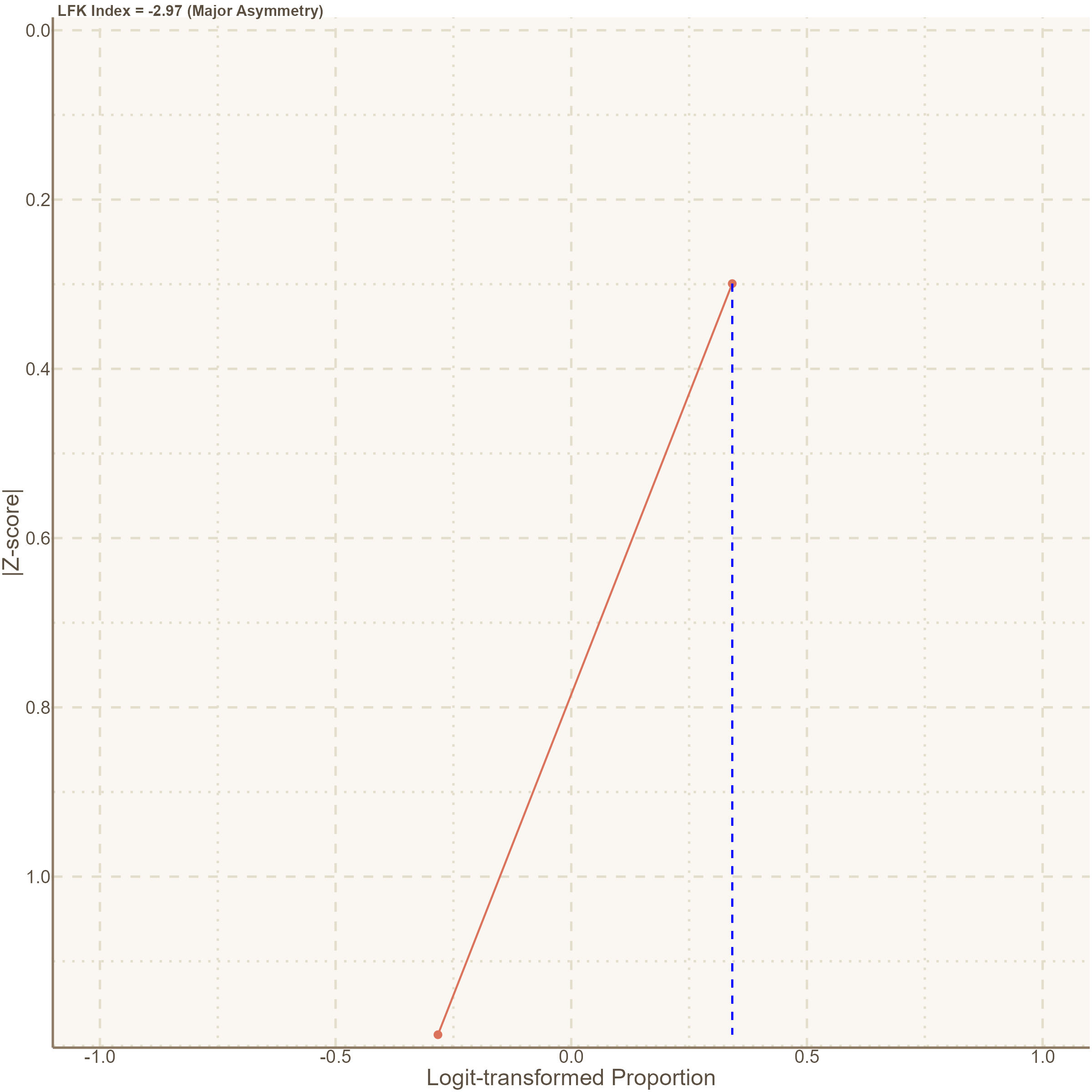

Supplement: Das et al. supplementary material [file S0950268824000177sup001.zip › S0950268824000177sup014.png]

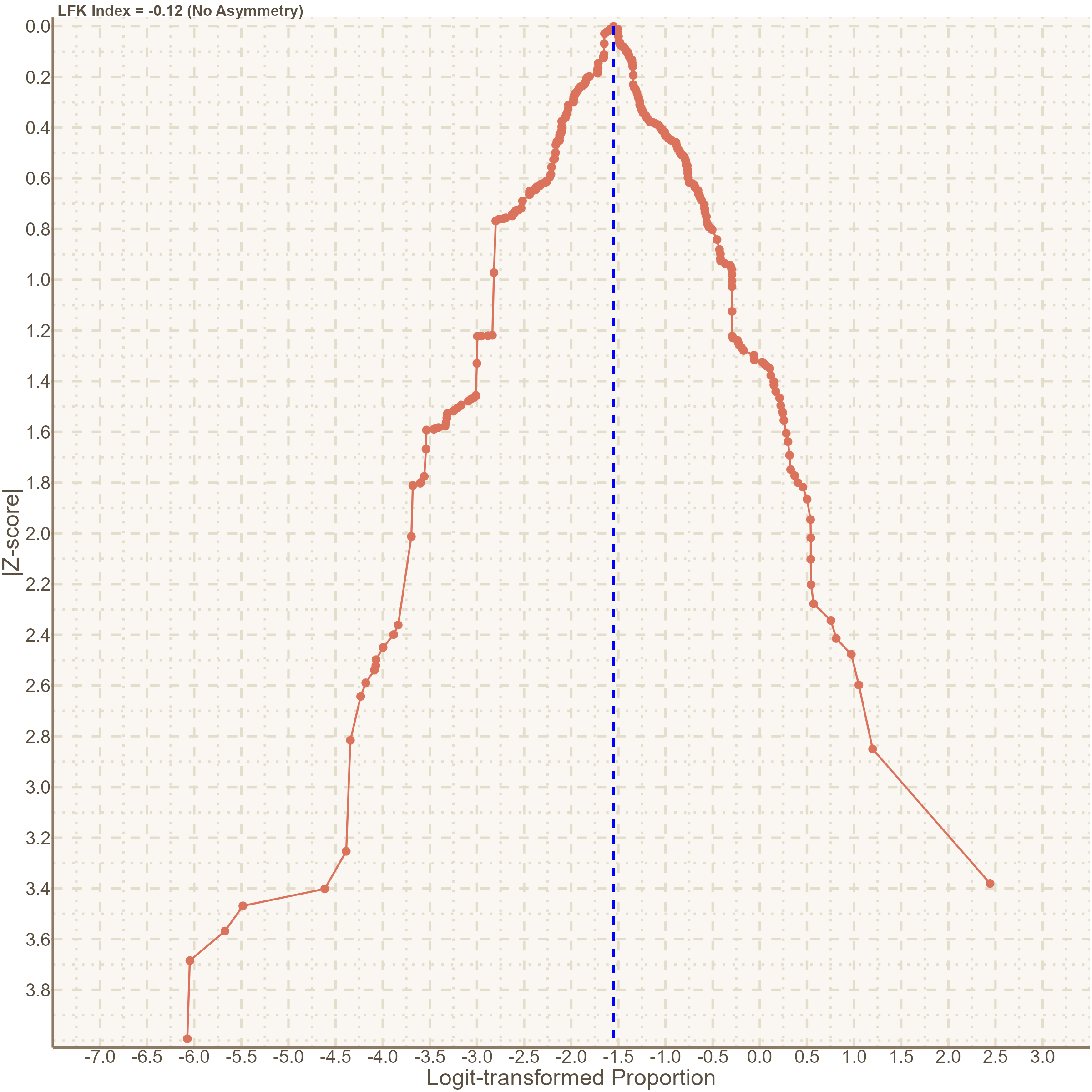

Supplement: Das et al. supplementary material [file S0950268824000177sup001.zip › S0950268824000177sup015.png]

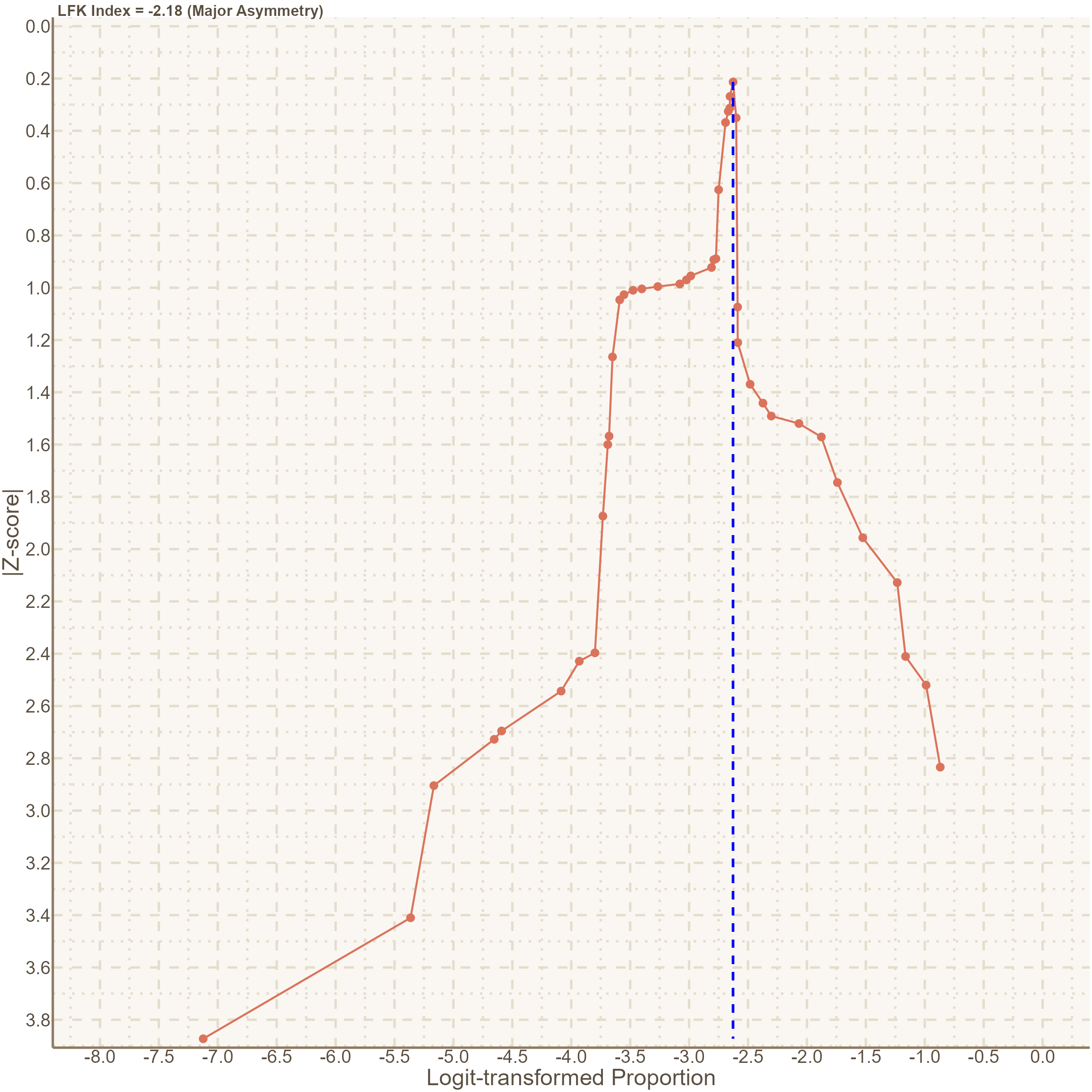

Supplement: Das et al. supplementary material [file S0950268824000177sup001.zip › S0950268824000177sup016.png]

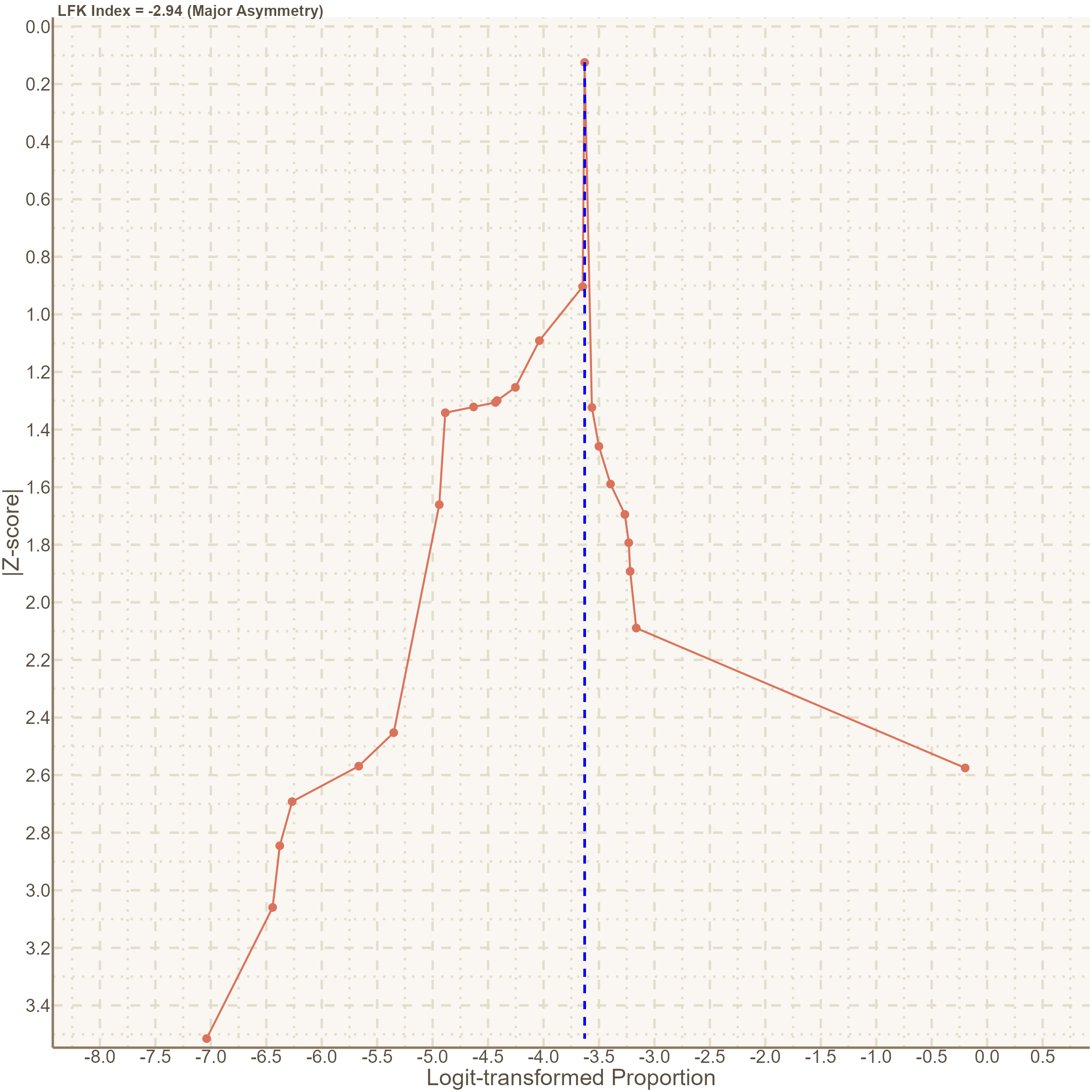

Supplement: Das et al. supplementary material [file S0950268824000177sup001.zip › S0950268824000177sup017.png]

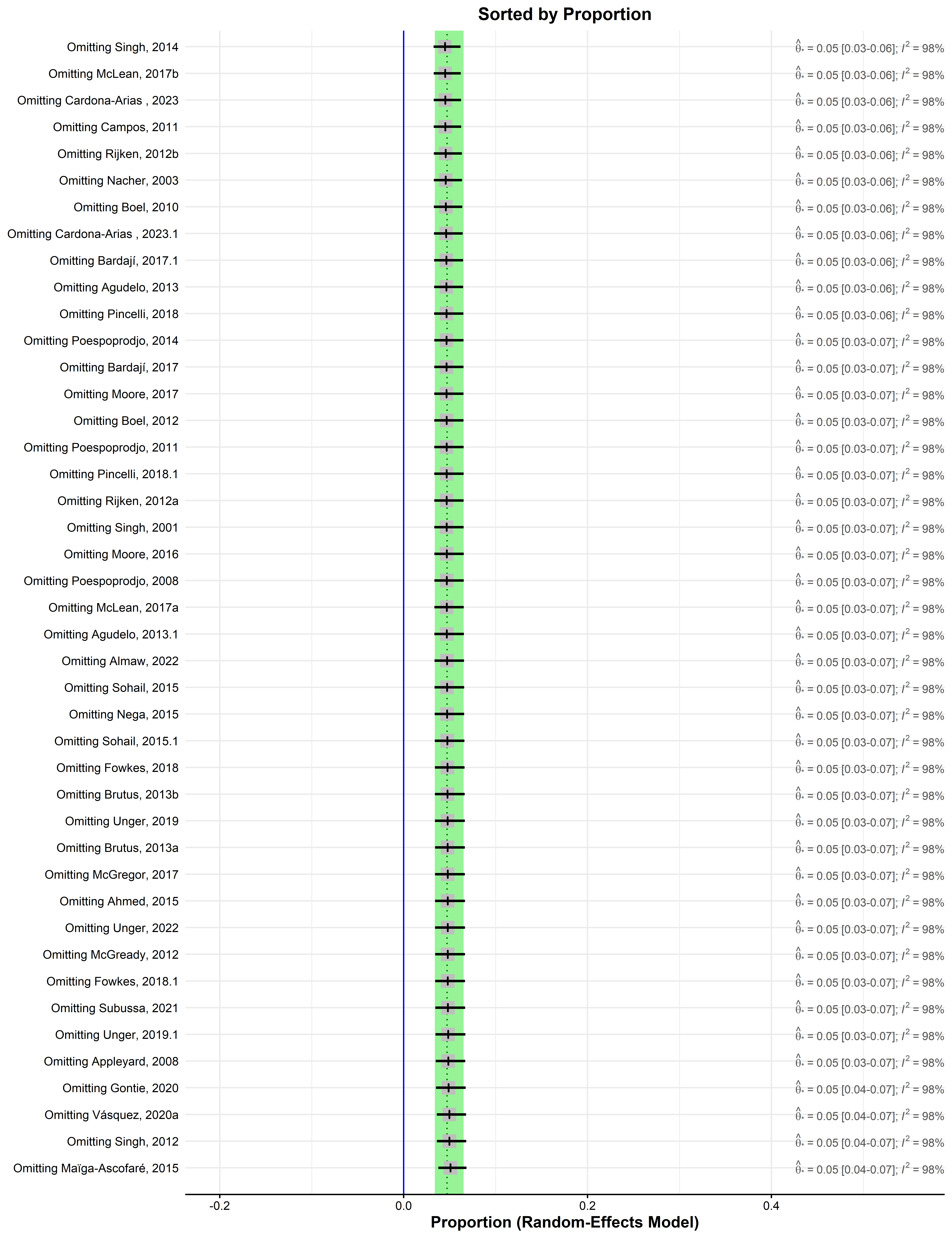

Supplement: Das et al. supplementary material [file S0950268824000177sup001.zip › S0950268824000177sup019.png]

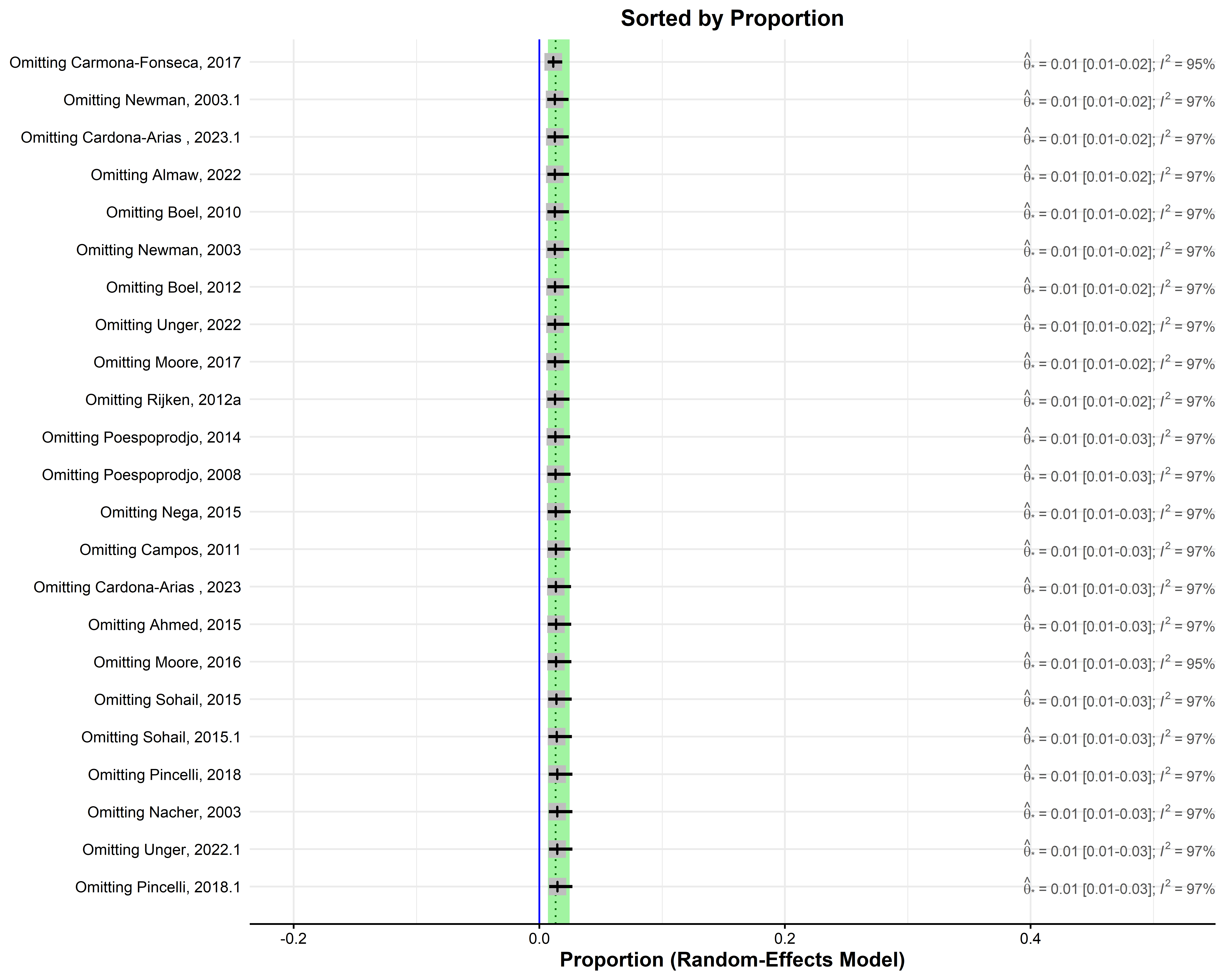

Supplement: Das et al. supplementary material [file S0950268824000177sup001.zip › S0950268824000177sup020.png]

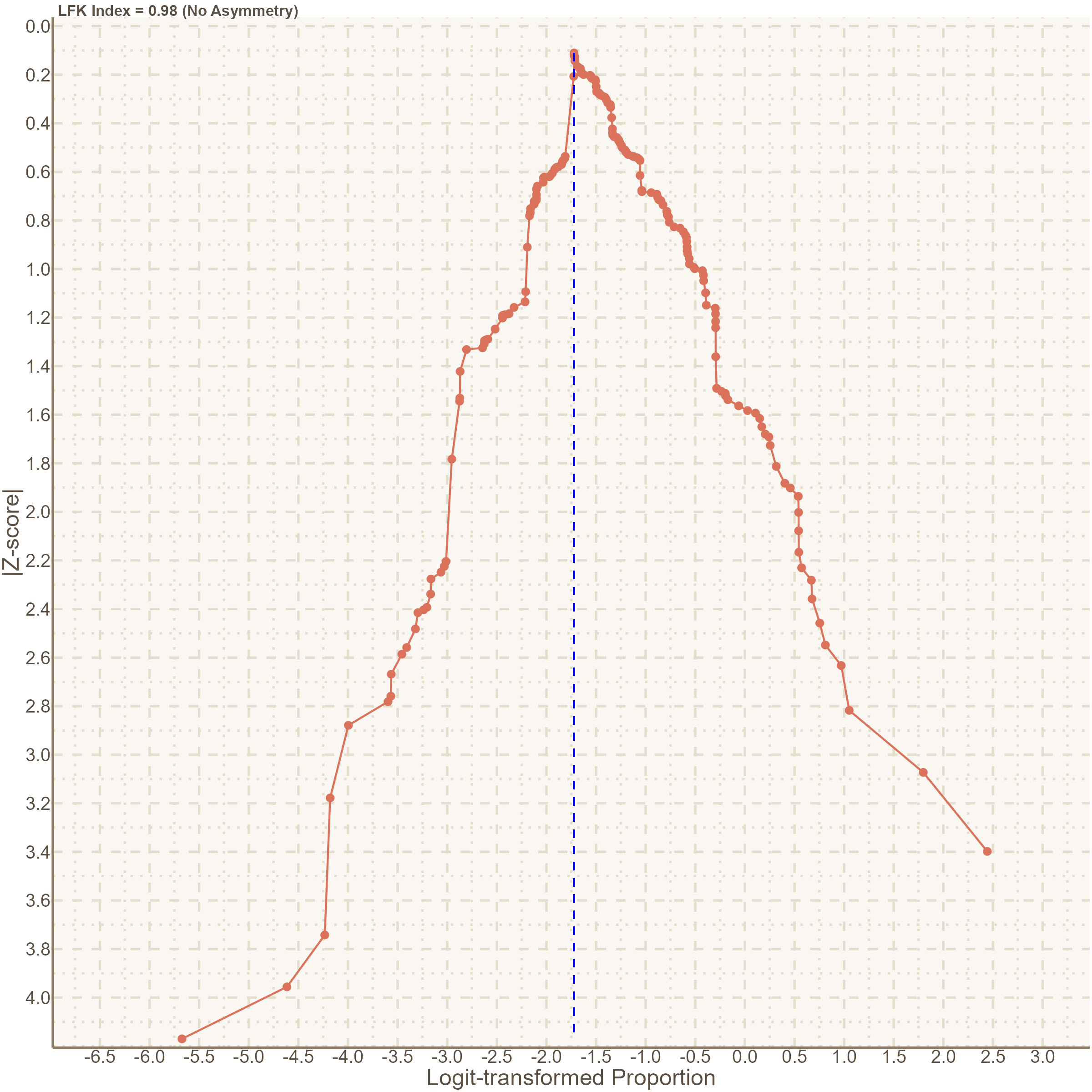

Supplement: Das et al. supplementary material [file S0950268824000177sup001.zip › S0950268824000177sup023.png]

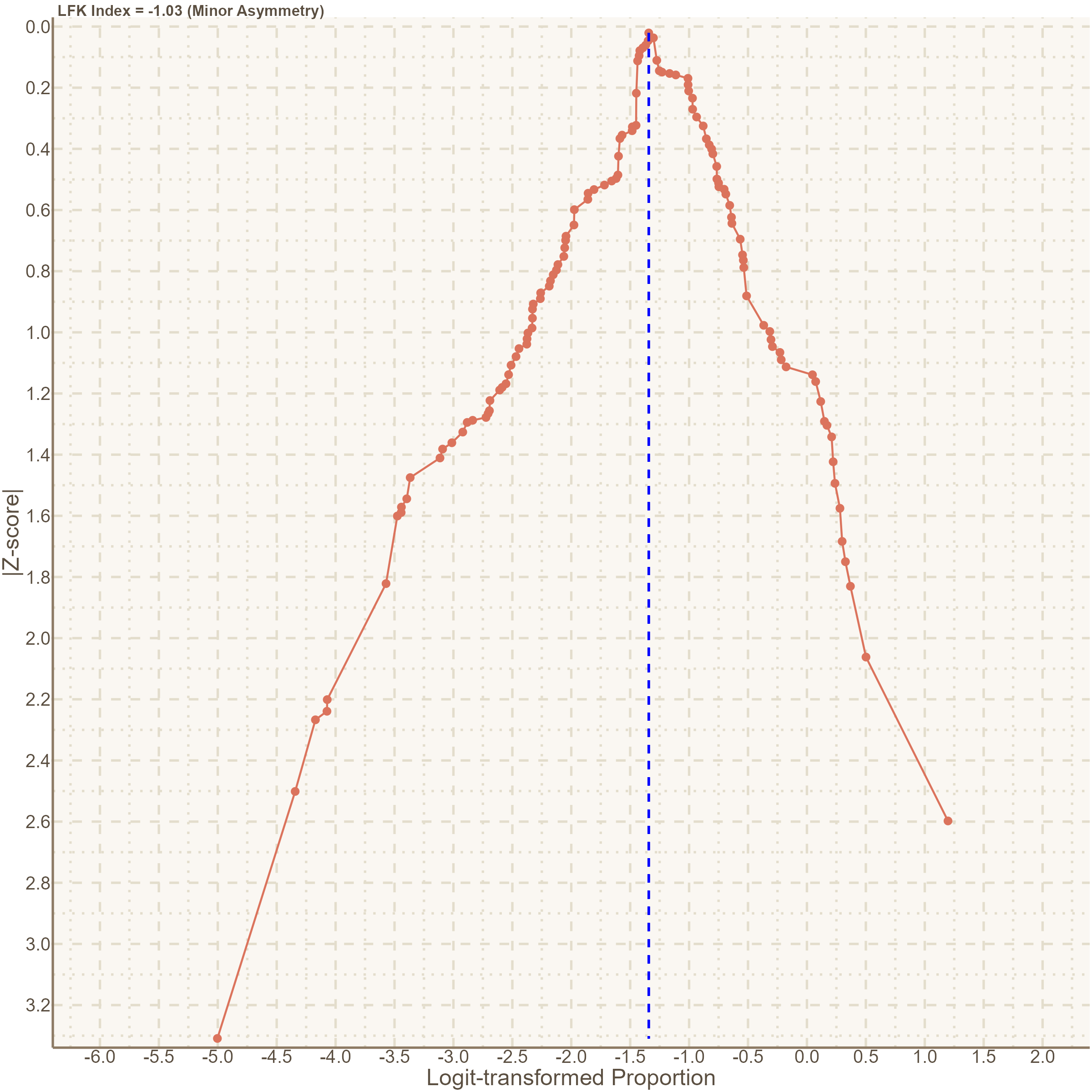

Supplement: Das et al. supplementary material [file S0950268824000177sup001.zip › S0950268824000177sup024.png]

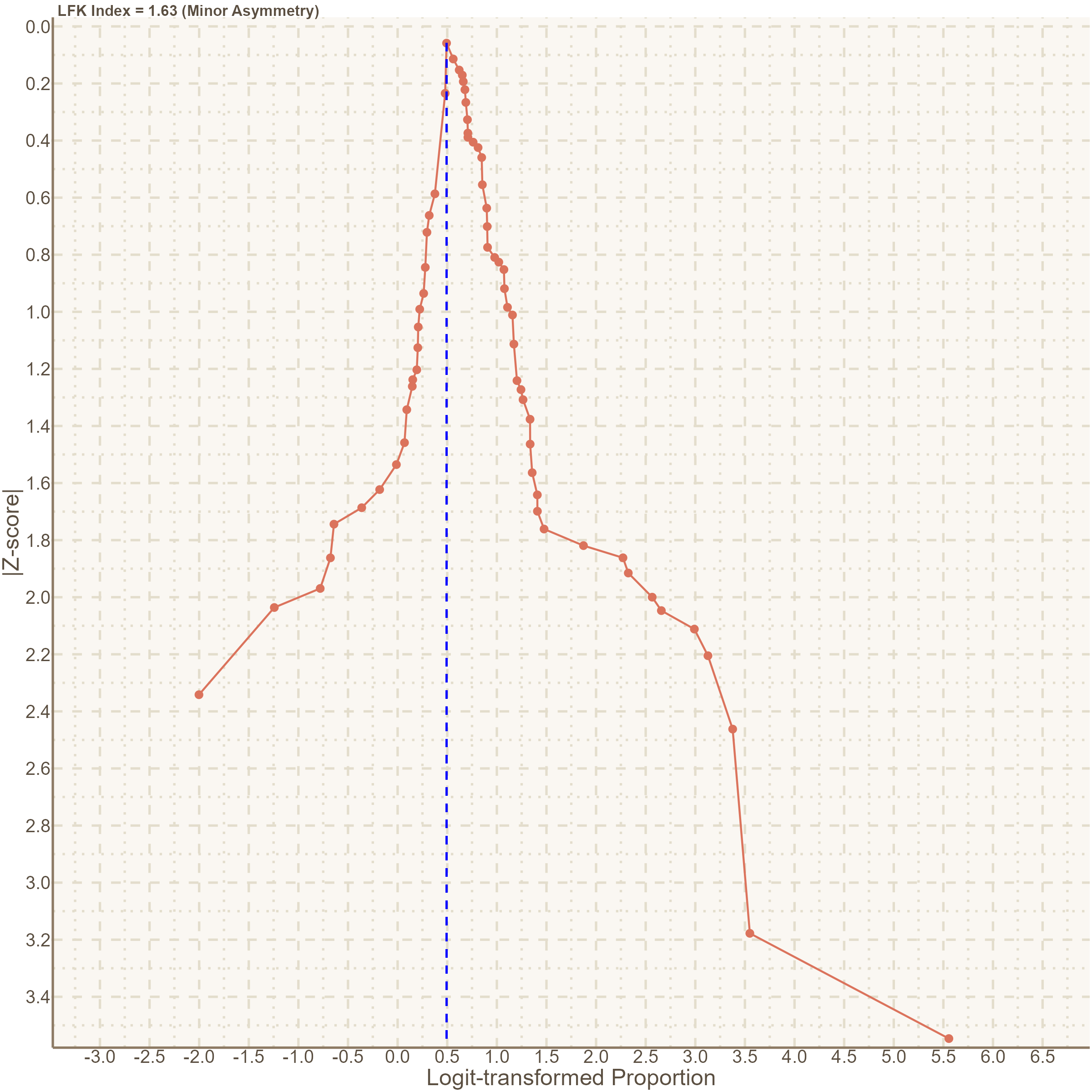

Supplement: Das et al. supplementary material [file S0950268824000177sup001.zip › S0950268824000177sup026.png]

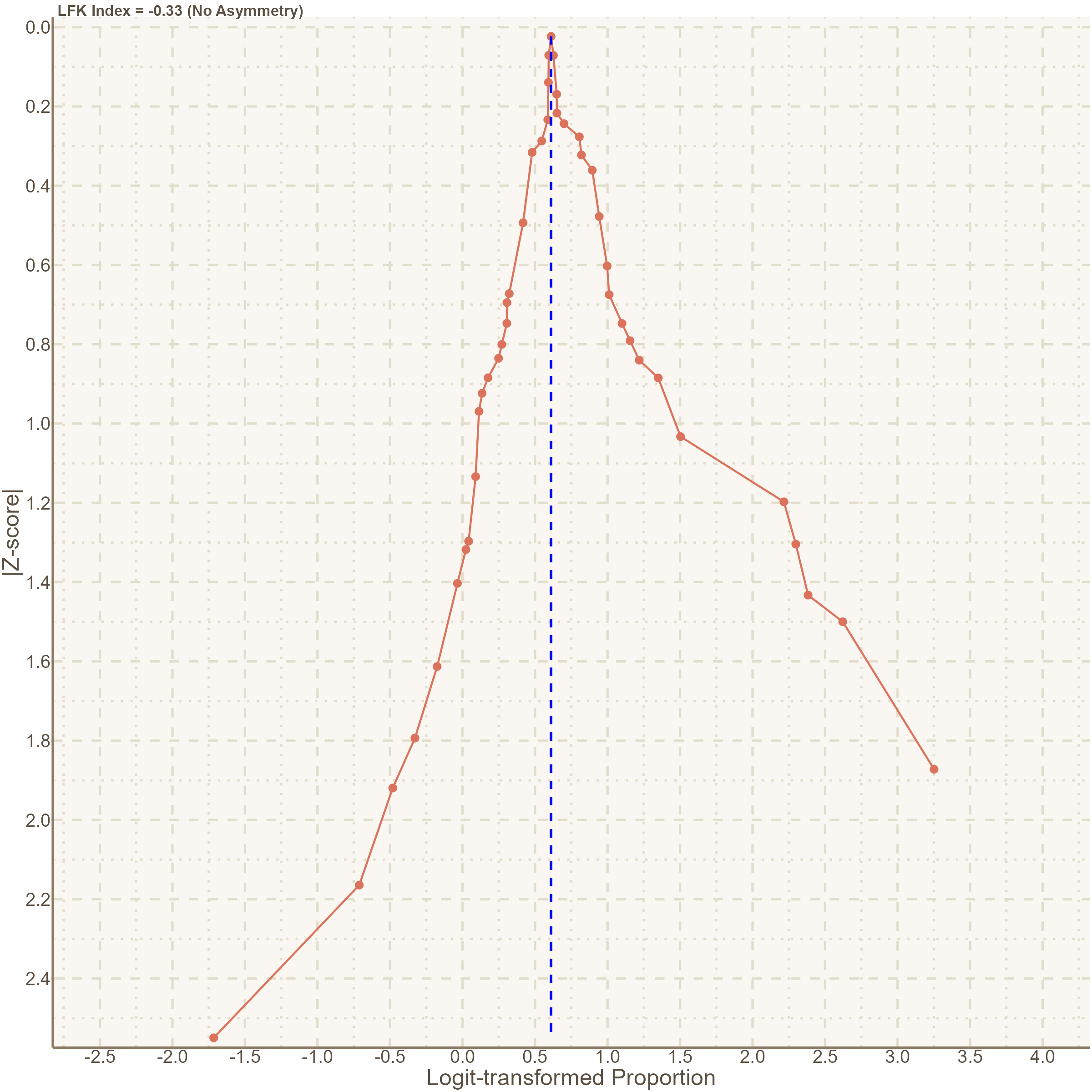

Supplement: Das et al. supplementary material [file S0950268824000177sup001.zip › S0950268824000177sup028.png]
